# Spatial organization of AQP4 water channel expression in the human brain

**DOI:** 10.1126/sciadv.aeg8117

**Published:** 2026-09-23

**Authors:** Tahmineh Taheri, Asa Farahani, Zhen-Qi Liu, Eric G. Ceballos, Adil Harroud, Alain Dagher, Bratislav Misic

**Affiliations:** ^1^ Montréal Neurological Institute, McGill University, Montréal, Québec, Canada.; ^2^ Department of Psychiatry, University of Cambridge, Cambridge, UK.; ^3^ Wolfson College, University of Cambridge, Cambridge, UK.

## Abstract

Aquaporin-4 (AQP4) water channels support the glymphatic system, a brain-wide pathway that clears cerebral waste products. Here, we use *AQP4* gene expression to reconstruct a whole-brain glymphatic-related topography and link it to vascular physiology, glioma, and vulnerability to neurodegenerative diseases. We find that *AQP4* expression is highly organized across the brain, peaking in subcortical, ventral, and periventricular territories, consistent with a clearance axis near cerebrospinal fluid reservoirs and perivascular interfaces. Linking *AQP4* expression to vascular organization, *AQP4*-enriched regions show lower normative blood perfusion, but lie close to areas commonly affected by cerebral small vessel disease. Turning to neurodegeneration, we find that atrophy patterns colocalize with *AQP4* expression, most strongly for tau and TDP-43 proteinopathies, and high-atrophy regions lie close to *AQP4* hotspots in anatomical and structural connectome space. Normative positron emission tomography markers of neuroinflammation strengthen the spatial alignment between *AQP4* expression and disease atrophy. Last, we show that glioma is most frequent in *AQP4*-enriched regions. Collectively, this work highlights how *AQP4* expression relates to brain physiology and vulnerability.

## INTRODUCTION

The brain is organized across multiple spatial scales (*1*). Genomic gradients (*2*, *3*) shape the spatial arrangement of cell types and laminar differentiation (*4*–*7*), which in turn determine the wiring of neural circuits and the emergence of patterned neural activity (*8*–*13*). This complex system is perpetually active, requiring constant exchange of fluids to deliver resources (such as glucose and oxygen) and clear waste products (such as metabolites and proteins) (*14*). While the former is relatively well studied, including metabolism and blood flow (*15*, *16*), the latter—known as the glymphatic system—(*17*) is comparatively less understood.

Central to the glymphatic system are aquaporin-4 (AQP4) integral membrane channel proteins (*18*–*20*). AQP4 channels are located primarily on astrocyte endfeet that line perivascular spaces, supporting the exchange of cerebrospinal fluid (CSF) and interstitial fluid (ISF) (*21*). The glymphatic system, powered by AQP4 water channels, drives bulk transport of CSF from the subarachnoid space along periarterial spaces; after mixing with ISF in the parenchyma, it exits the parenchyma through perivenous spaces (*19*, *22*, *23*). This process regulates directional ISF movement, waste clearance, and brain health. Despite its importance, how AQP4 is organized at the whole-brain level is unknown.

The spatial arrangement of AQP4 channels is an important question, as multiple neurological diseases are associated with AQP4 dysfunction (*24*, *25*). Neuromyelitis optica spectrum disorder (NMOSD) is an autoimmune disease in which AQP4 antibodies bind to the channel, causing inflammation and disrupting AQP4 function (*26*–*28*). NMOSD brain lesions show preferential involvement of regions with high *AQP4* expression (*29*). Beyond NMOSD, AQP4-supported perivascular fluid exchange is relevant to cerebral small vessel disease (CSVD). Glymphatic dysfunction has been proposed as a mechanism linking impaired perivascular fluid transport with CSVD, a condition commonly marked by white matter hyperintensities (WMHs) (*30*–*33*). AQP4 dysfunction is also implicated in neurodegenerative diseases. The loss of perivascular AQP4 polarization accompanies aging and neurodegeneration, with potential consequences for interstitial solute clearance and the accumulation of misfolded proteins such as amyloid-β, tau, and TDP-43 (*34*–*38*). Moreover, AQP4 has been implicated in glioma biology. *AQP4* expression is altered in glioblastoma and peritumoral tissue, and experimental studies suggest that AQP4 can contribute to glioma cell migration, invasion, and infiltration into surrounding tissue (*39*–*41*). As the main astrocytic water channel, AQP4 is also relevant to water handling in the peritumoral environment (*42*–*44*). Neuroinflammation may exacerbate these processes by altering astrocyte reactivity and further disrupting perivascular AQP4 polarization, creating a cycle in which inflammation, glymphatic dysfunction, impaired waste clearance, and neurodegeneration reinforce each other (*20*, *45*, *46*). Together, AQP4 supports healthy brain waste clearance and water balance, and its dysfunction is linked with regional vulnerability to multiple neurological diseases.

Here, we study the whole-brain organization of *AQP4*. We first map the expression of *AQP4* gene in cortex and subcortex and use this map as a molecular anchor for the glymphatic system. Looking at healthy brain function, we ask how the spatial distribution of *AQP4* relates to regional differences in blood perfusion and vein density. Looking at dysfunction, we investigate how the spatial distribution of *AQP4* relates to regional differences in vulnerability to CSVD, neurodegenerative disease, and glioma. We also ask whether normative positron emission tomography (PET) markers of neuroinflammation improve these associations. Last, we assess the extent to which these results are specific to *AQP4* by repeating key analyses for related aquaporins, AQP1 and AQP9.

## RESULTS

### Mapping AQP4 in the brain

AQP4 monomers are encoded by the *AQP4* gene. *AQP4* expression is estimated across the brain using microarray gene expression data from the Allen Human Brain Atlas (AHBA) (*2*). Quality control includes intensity-based filtering and differential stability analysis (*47*) (see Materials and Methods). Expression values are parcellated to the Schaefer 400 cortical (*48*) and Melbourne subcortical S4 (*49*) atlases, yielding a whole-brain parcellation of 454 regions. To validate the spatial distribution of AHBA microarray measurements, we compare it with RNA-seq data from AHBA (fig. S1A) and the Human Protein Atlas (HPA) as an independent dataset (fig. S1B) (*50*, *51*). We also validate the *AQP4* map against independent mass spectrometry proteomics data, showing a positive association with AQP4 protein abundance (fig. S2) (*52*). Moreover, *AQP4* expression is positively correlated with MLC1 expression, a membrane protein enriched in astrocytes and localized to perivascular endfeet at blood-brain and CSF-brain interfaces (*53*, *54*). This association provides additional support that *AQP4* expression map captures spatial variation related to perivascular astrocytic organization (fig. S3). In addition, we compare *AQP4* expression with an in vivo tracer assay of glymphatic transport. We use regional brain enrichment of an intrathecally administered magnetic resonance imaging (MRI) tracer (gadobutrol) measured 24 hours after administration in a previously reported reference cohort (*55*) (see Materials and Methods). *AQP4* expression shows spatial correspondence with the 24-hour tracer enrichment map (fig. S4), supporting *AQP4* topography as a molecular proxy for glymphatic organization in humans.

**Fig. 1.**
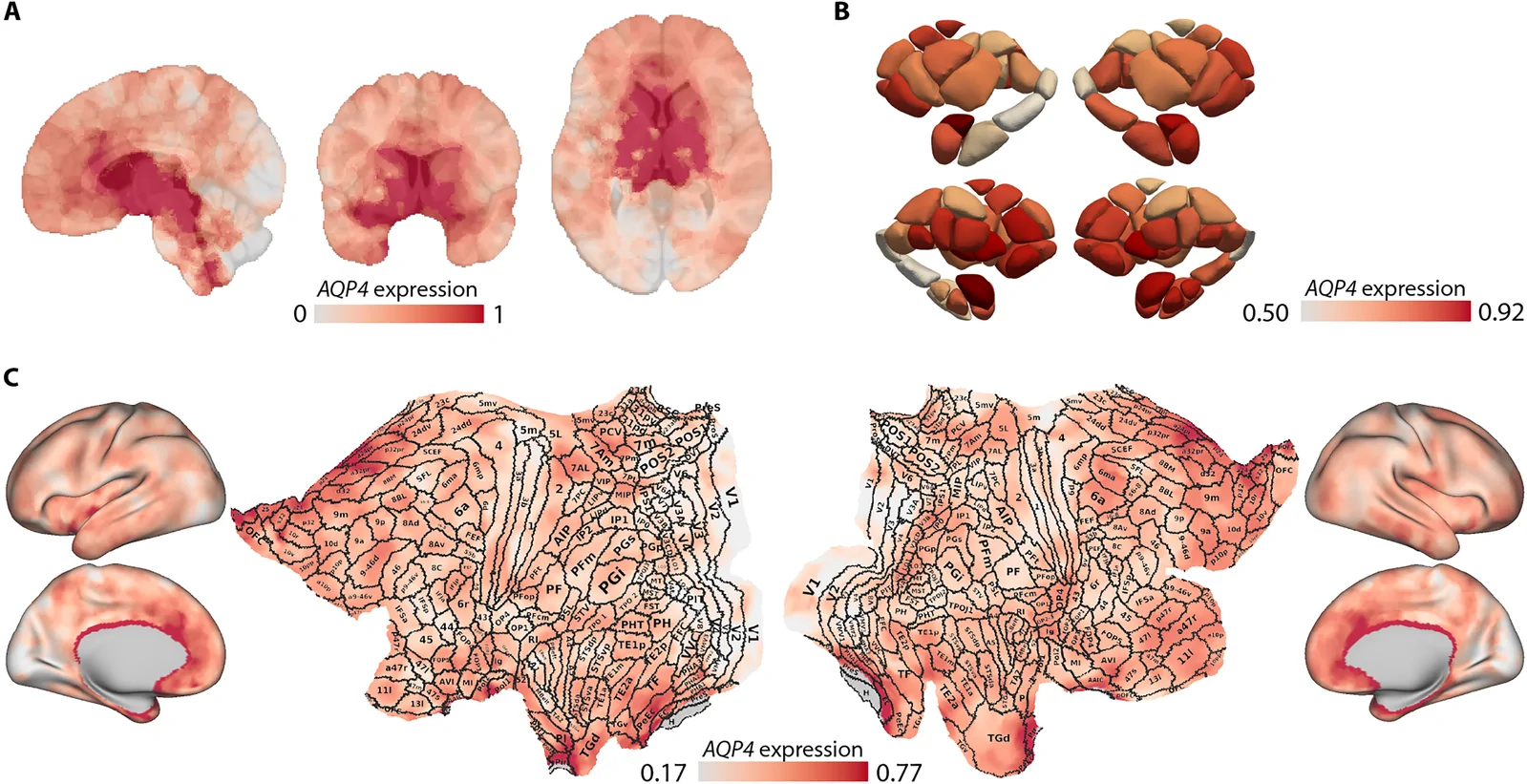
Whole-brain, cortical, and subcortical *AQP4* expression maps. Donor-averaged *AQP4* expression from the AHBA (*2*) processed with the abagen workflow (see Materials and Methods) (*47*). (**A**) A dense, interpolated volumetric *AQP4* expression map is displayed on a T1-weighted MNI template in sagittal, coronal, and axial views (MNI152-NonLinear2009cAsym, 1 mm; slice indices: sagittal = 102, coronal = 137, axial = 81). (**B**) Subcortical *AQP4* expression parcellated to the Melbourne subcortical S4 atlas (*49*), shown as 3D parcel surfaces across multiple views. Subcortical parcel names and abbreviations are listed in table S1; labeled renderings are shown in fig. S10. (**C**) Cortical *AQP4* expression is shown on fsLR inflated and flat surfaces by projecting the dense volumetric map to the cortical surface; borders and areal labels from the multimodal Glasser parcellation are overlaid on the flat surface for anatomical reference (*169*); the full Glasser nomenclature is provided in table S2.

Figure 1A shows the spatial pattern of *AQP4* expression across the brain. *AQP4* is expressed throughout the brain, with greater expression near subcortical, periventricular, and ventral regions. This periventricular enrichment is also evident in a volume rendering of high *AQP4* expression values (fig. S5). Within the subcortex, *AQP4* expression is highest in amygdala and basal ganglia territories, including pallidum, caudate, putamen, and nucleus accumbens, and is also elevated in ventral thalamic nuclei. In contrast, hippocampal regions show lower expression overall, with the lowest values in posterior subdivisions (Fig. 1B). In the cortex, we find greater *AQP4* expression throughout the limbic and transmodal cortices, including cingulate cortex, insula, medial temporal lobe, and medial prefrontal cortex. Conversely, *AQP4* expression is lowest in unimodal cortex, such as primary visual, somatosensory, and auditory cortex (Fig. 1C). In sum, the expression of *AQP4* is enriched in periventricular, ventral, and subcortical regions, elevated across limbic cortex, and lowest in primary sensory areas. In the following sections, we investigate the extent to which this organization aligns with other related systems (*56*).

### AQP4 channels and vascular organization

**Fig. 2.**
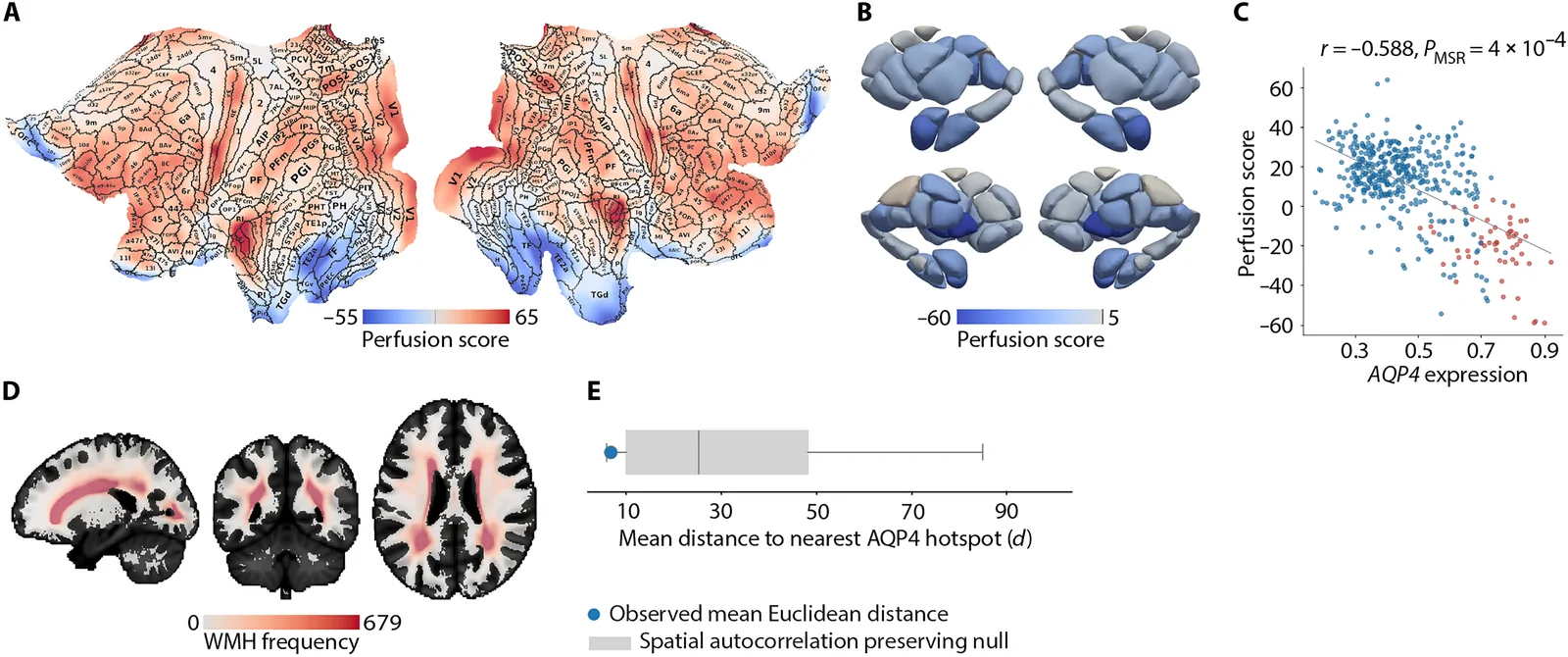
*AQP4* expression relates to vascular organization. (**A**) Cortical cerebral blood perfusion (PC1), derived from the HCP Lifespan dataset (*58*–*61*), is shown on a 2D flat fsLR surface. Borders and areal names from the multimodal Glasser parcellation are overlaid on the flat surface (*169*); the full Glasser nomenclature is provided in table S2. (**B**) Subcortical blood perfusion parcellated to the Melbourne subcortical S4 atlas (*49*) and shown as 3D parcel surfaces across multiple views. Subcortical parcel names and abbreviations are listed in table S1, and labeled parcel renderings are shown in fig. S10. (**C**) Pearson correlation between *AQP4* expression from the Allen Human Brain Atlas and blood perfusion (r=−0.588, $P_{\text{MSR}}=4\times 10^{-4}$). Each point is a parcel [blue: Schaefer 400 cortical parcels (*48*); red: Melbourne S4 subcortical parcels (*49*)]. The gray line shows the least-squares fit. $P_{\text{MSR}}$ is computed using Moran spectral randomization ($N_{\text{MSR}}=10,000$) (*71*) (see Materials and Methods). (**D**) Volumetric WMH frequency map from a multicenter memory-clinic dataset (*N* = 3525) is displayed on a T1-weighted MNI template in sagittal, coronal, and axial views (MNI152-NonLinear2009cAsym, 1 mm; slice indices: sagittal = 110, coronal = 76, axial = 96) (*64*). (**E**) Observed mean distance is shown as a point relative to a spatial autocorrelation-preserving null distribution generated with Moran spectral randomization ($N_{\text{MSR}}=10,000$).

Given the close anatomical relationship between glymphatic transport and the cerebrovascular system, including the localization of AQP4 channels at astrocytic endfeet surrounding blood vessels (*21*, *24*, *57*), we test whether the spatial pattern of *AQP4* expression covaries with regional cerebral blood perfusion, a macroscale measure of blood supply. To address this question, we estimate cerebral blood perfusion using pseudo-continuous arterial spin labeling (ASL) MRI and derive a normative perfusion map in the HCP Lifespan dataset (1305 participants; Fig. 2, A and B; see Materials and Methods) (*58*–*61*). We find that *AQP4* expression and blood perfusion are significantly anticorrelated (Fig. 2C; r=−0.588, $P_{\text{MSR}}=4\times 10^{-4}$), indicating that regions with higher *AQP4* expression tend to show lower normative perfusion. We observe a similar negative correlation between *AQP4* expression and vein density using the Venous Neuroanatomy (VENAT) atlas, derived from high-resolution 7-T quantitative susceptibility mapping (QSM) in healthy adults (fig. S6C; r=−0.296, $P_{\text{MSR}}=0.005$) (*62*). These findings suggest that higher perfusion and vein density may reflect larger vessels with lower *AQP4* expression.

In addition to these macroscale vascular measures, we next ask whether *AQP4*-enriched regions are spatially related to WMH, a major marker of CSVD. This analysis is motivated by the perivascular localization of AQP4 and by growing work linking glymphatic clearance to small vessel disease. AQP4 is concentrated at astrocytic endfeet surrounding perivascular spaces, where it supports CSF-ISF exchange. Impairment of this perivascular clearance pathway has been proposed to contribute to CSVD, and MRI-based glymphatic clearance measures have been associated with WMH burden, lacunes, microbleeds, and enlarged perivascular spaces (*30*–*32*, *63*). Here, we use a group-level WMH frequency map from the Meta VCI Map Consortium memory-clinic cohort (3525 participants; see Materials and Methods) (*64*). The map captures the spatial distribution of WMH and shows the expected concentration of lesions in periventricular white matter (Fig. 2D). For each high-WMH region, we compute the Euclidean distance to the nearest *AQP4* hotspot and average distances across regions, then assess significance using Moran spectral randomization (MSR). We find that high-WMH regions are closer to *AQP4* hotspots than expected under the spatial null model (Fig. 2E; *d* = 5.93; $P_{\text{MSR}}=0.037$). This spatial proximity suggests that *AQP4*-enriched regions occupy a topography close to regions commonly affected by small vessel–related white matter injury.

### AQP4 channels and susceptibility to neurodegeneration

Neurodegenerative diseases are characterized by the accumulation of misfolded, aggregate-prone proteins in the brain, including amyloid-β, tau, and TDP-43 (*65*, *66*). Because these proteins are cleared in part through AQP4-mediated perivascular fluid transport, regional differences in *AQP4* expression may be associated with where pathology occurs. One possibility is that regions with higher *AQP4* expression may be more dependent on intact AQP4-mediated perivascular transport and, therefore, more vulnerable when AQP4 localization or function is disrupted. This hypothesis is motivated by studies showing that, in aging and neurodegenerative disease, AQP4 is frequently mislocalized, with reduced perivascular polarization at astrocyte endfeet. Such changes can impair CSF-ISF exchange and slow the clearance of interstitial solutes (*24*, *34*, *36*, *38*, *67*). Slower clearance may increase the residence time of soluble proteins in the extracellular space, potentially contributing to protein accumulation and neurodegeneration. A second, nonexclusive possibility is that *AQP4*-enriched regions may mark territories where perivascular solute transport is especially relevant to long-term vulnerability. Because our data measure regional *AQP4* expression rather than AQP4 polarization or glymphatic flux, we treat these possibilities as hypotheses and test whether *AQP4* expression spatially aligns with disease-related atrophy.

To test this idea at the whole-brain level, we ask whether the spatial distribution of *AQP4* relates to patterns of gray matter loss across neurodegenerative diseases. We use voxel-based morphometry (VBM) maps from a pathologically confirmed dementia cohort (*68*) comprising early-onset Alzheimer’s disease (EOAD), late-onset Alzheimer’s disease (LOAD), presenilin 1 mutation carriers (PS1), three-repeat tauopathy (3Rtau), four-repeat tauopathy (4Rtau), frontotemporal lobar degeneration with TDP-43 type A pathology (TDP-43A), frontotemporal lobar degeneration with TDP-43 type C pathology (TDP-43C), and dementia with Lewy bodies (DLB) (see Materials and Methods). Atrophy is estimated using VBM applied to the T1-weighted images, which is then parcellated to the same Schaefer-Melbourne atlas as the *AQP4* map (*48*, *49*). We then correlate *AQP4* expression with each atrophy pattern and assess significance using MSR to account for spatial autocorrelation (*69*–*71*) (see Materials and Methods).

**Fig. 3.**
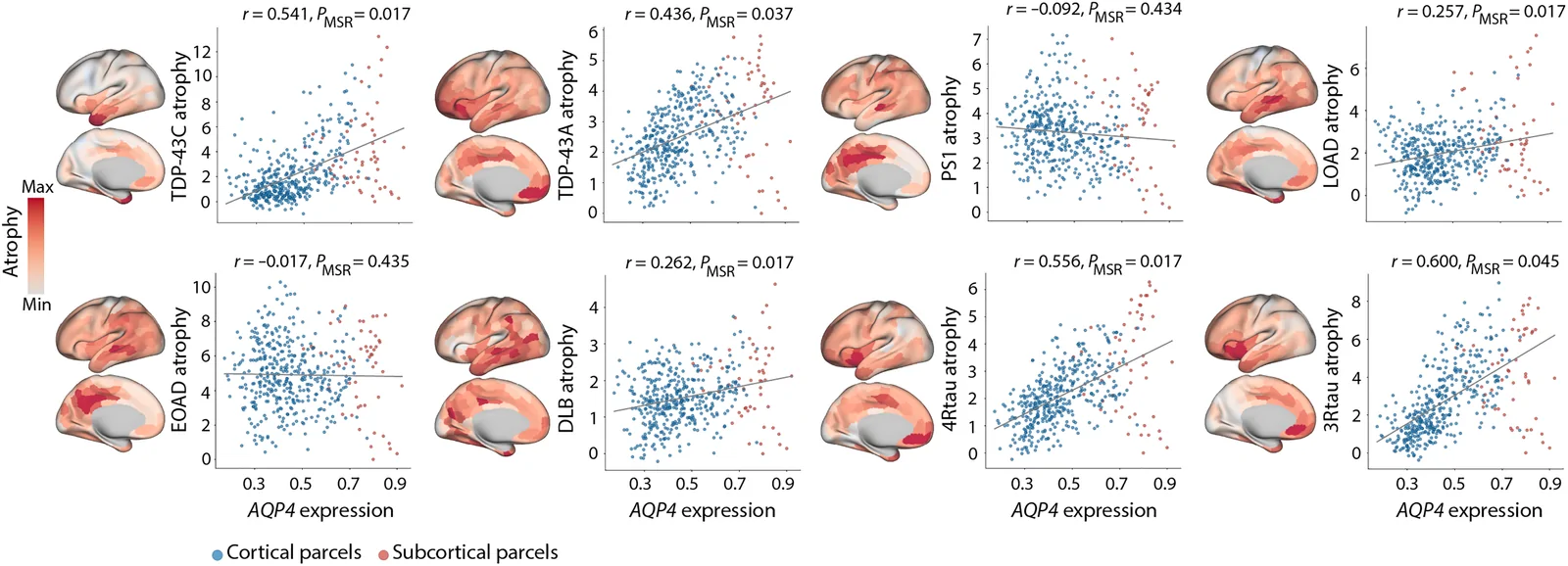
Colocalization of *AQP4* expression with dementia atrophy patterns. Insets show cortical atrophy maps on left hemisphere fsLR surfaces. Scatterplots show parcel-wise Pearson correlations between *AQP4* expression (*x* axis) and regional atrophy (*y* axis) from a pathologically confirmed dementia cohort (*68*). Panels (left to right) are as follows: frontotemporal lobar degeneration with TDP-43 type C pathology (TDP-43C; *r* = 0.541; FDR-corrected, $P_{\text{MSR}}=0.017$), frontotemporal lobar degeneration with TDP-43 type A pathology (TDP-43A; *r* = 0.436; FDR-corrected, $P_{\text{MSR}}=0.037$), presenilin 1 mutation carriers (PS1; r=−0.092; FDR-corrected, $P_{\text{MSR}}=0.434$), late-onset Alzheimer’s disease (LOAD; *r* = 0.257; FDR-corrected, $P_{\text{MSR}}=0.017$), early-onset Alzheimer’s disease (EOAD; r=−0.017; FDR-corrected, $P_{\text{MSR}}=0.435$), dementia with Lewy bodies (DLB; *r* = 0.262; FDR-corrected, $P_{\text{MSR}}=0.017$), four-repeat tauopathy (4Rtau; *r* = 0.556; FDR-corrected, $P_{\text{MSR}}=0.017$), and three-repeat tauopathy (3Rtau; *r* = 0.600; FDR-corrected, $P_{\text{MSR}}=0.045$). Each point is a parcel (blue: cortical parcels; red: subcortical parcels). Gray lines show least-squares fits. $P_{\text{MSR}}$ values are computed using Moran spectral randomization ($N_{\text{MSR}}=10,000$).

Across diseases, we observe differences in how strongly atrophy colocalizes with *AQP4* expression (Fig. 3). TDP-43 and tau proteinopathies with similar patterns of frontotemporal volume loss show the strongest positive correlations with *AQP4* expression, including TDP-43C (*r* = 0.541; FDR-corrected, $P_{\text{MSR}}=0.017$), TDP-43A (*r* = 0.436; FDR-corrected, $P_{\text{MSR}}=0.037$), 4Rtau (*r* = 0.556; FDR-corrected, $P_{\text{MSR}}=0.017$), and 3Rtau (*r* = 0.600; FDR-corrected, $P_{\text{MSR}}=0.045$). These pathologies are characterized by marked atrophy of frontal, insular, and anterior temporal cortices, regions in which *AQP4* expression is also relatively high. More modest but still significant associations are observed for LOAD (*r* = 0.257; FDR-corrected, $P_{\text{MSR}}=0.017$) and DLB (*r* = 0.262; FDR-corrected, $P_{\text{MSR}}=0.017$), whose atrophy patterns emphasize medial temporal and temporoparietal regions. By contrast, EOAD and PS1 mutation carrier groups show weak and nonsignificant correlations with *AQP4* expression (both ∣r∣<0.1; FDR-corrected, $P_{\text{MSR}}>0.4$), consistent with their more extensive temporoparietal and sensorimotor volume loss, which only partially overlaps with the *AQP4* gradient. These results suggest that atrophy is spatially aligned with regions of higher *AQP4* expression.

### Proximity of AQP4 hotspots to high-atrophy regions

**Fig. 4.**
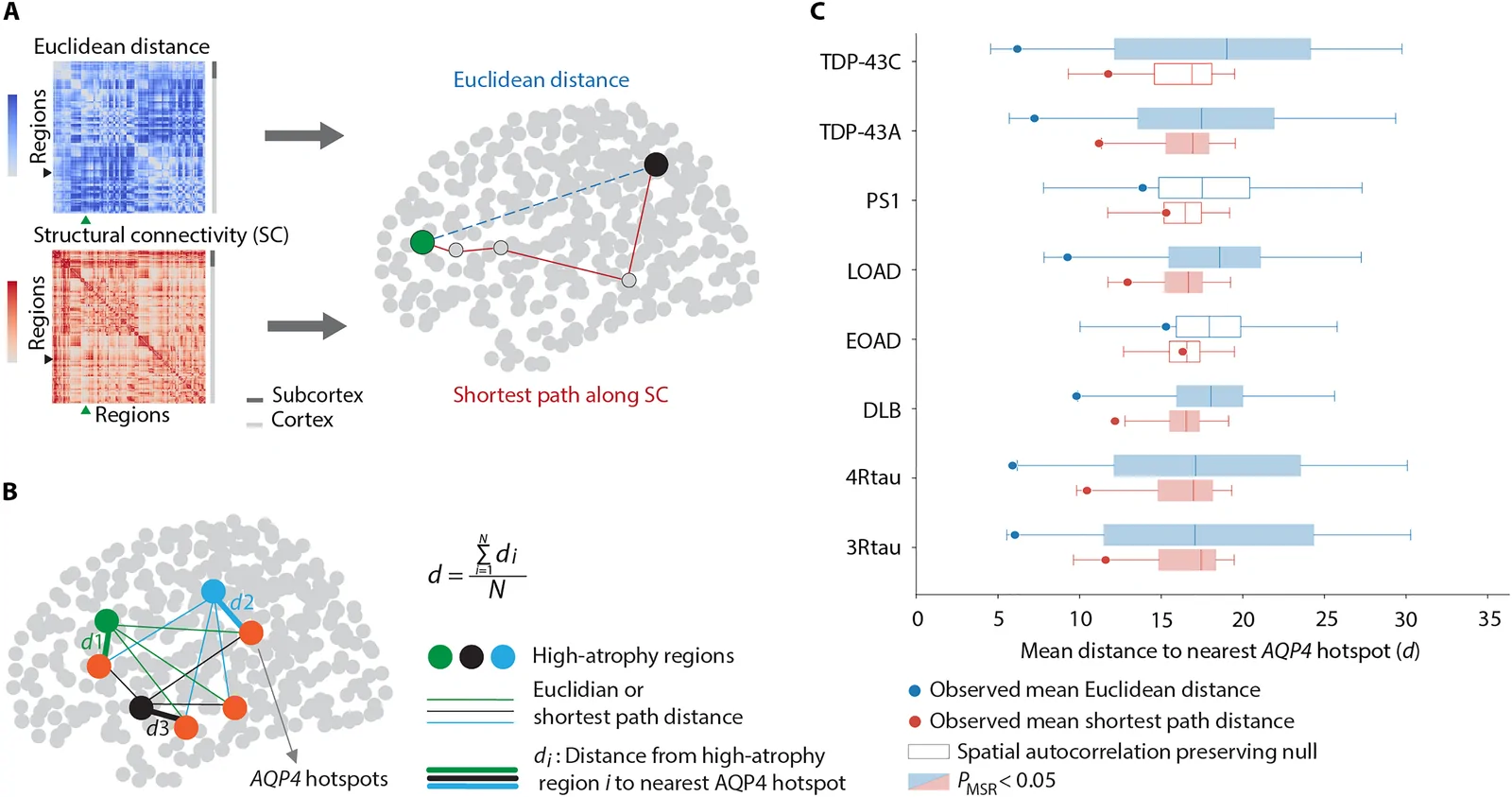
Proximity of high-atrophy regions to *AQP4* hotspots in anatomical and network space. (**A**) Schematic contrasting Euclidean distance in anatomical space with shortest path distance along the SC. (**B**) Schematic of the proximity analysis. For each disease, high-atrophy regions are parcels in the top decile of disease-related atrophy, and *AQP4* hotspots are parcels in the top decile of *AQP4* expression. For each high-atrophy parcel, we compute the distance to its nearest *AQP4* hotspot using both metrics and average across high-atrophy parcels to obtain a mean distance (*d*) per disease. The SC is computed from the HCP S1200 release (*N* = 1065; see Materials and Methods) (*77*). (**C**) Observed mean distances (points; blue: Euclidean, red: SC shortest path) are shown relative to spatial autocorrelation–preserving null distributions generated with Moran spectral randomization ($N_{\text{MSR}}=10,000$). Filled boxplots indicate significant proximity to *AQP4* hotspots (FDR-corrected, $P_{\text{MSR}}<0.05$). Disease abbreviations match Fig. 3.

So far, we have shown that *AQP4* expression is associated with disease-specific atrophy patterns across multiple neurodegenerative diseases. Here, we ask whether the most affected regions lie proximal to *AQP4*-enriched territories. To address this question, we define *AQP4* hotspots as parcels in the top decile of *AQP4* expression and high-atrophy regions as parcels in the top decile of atrophy. For each high-atrophy parcel, we compute the distance to its nearest *AQP4* hotspot using two metrics: (i) Euclidean distance in anatomical space and (ii) shortest path distance along the structural connectome (SC), motivated by empirical findings and computational models suggesting that neurodegenerative vulnerability propagates along white-matter connections (*72*–*76*) (Fig. 4A). Shortest path distances are computed on a group-averaged SC derived from the Human Connectome Project (HCP) S1200 release (*N* = 1065; see Materials and Methods) (*77*). We then average distances across high-atrophy parcels to obtain a mean Euclidean and a mean shortest path distance for each disease, and assess significance using MSR (*71*) (Fig. 4B).

For all diseases except PS1 and EOAD, the mean Euclidean distance from high-atrophy parcels to the nearest *AQP4* hotspot is significantly reduced relative to the null distribution, indicating that atrophy tends to occur in the spatial neighborhood of *AQP4*-enriched territories. Spatial proximity (Euclidean distance) is significant for TDP-43C (*d* = 6.19; FDR-corrected, $P_{\text{MSR}}=11.5\times 10^{-3}$), TDP-43A (*d* = 7.24; FDR-corrected, $P_{\text{MSR}}=7.68\times 10^{-3}$), LOAD (*d* = 9.26; FDR-corrected, $P_{\text{MSR}}=7.68\times 10^{-3}$), DLB (*d* = 9.81; FDR-corrected, $P_{\text{MSR}}=2.93\times 10^{-3}$), 4Rtau (*d* = 5.89; FDR-corrected, $P_{\text{MSR}}=1.59\times 10^{-3}$), and 3Rtau (*d* = 6.04; FDR-corrected, $P_{\text{MSR}}=1.59\times 10^{-3}$), but not for PS1 (*d* = 13.86; FDR-corrected, $P_{\text{MSR}}=0.182$) or EOAD (*d* = 15.29; FDR-corrected, $P_{\text{MSR}}=0.182$) (Fig. 4C). We find similar results in network space. Shortest path proximity is significant for TDP-43A (*d* = 11.19; FDR-corrected, $P_{\text{MSR}}=0.036$), LOAD (*d* = 12.94; FDR-corrected, $P_{\text{MSR}}=0.048$), DLB (*d* = 12.17; FDR-corrected, $P_{\text{MSR}}=0.019$), 4Rtau (*d* = 10.45; FDR-corrected, $P_{\text{MSR}}=0.019$), and 3Rtau (*d* = 11.59; FDR-corrected, $P_{\text{MSR}}=0.048$), but not for TDP-43C (*d* = 11.75; FDR-corrected, $P_{\text{MSR}}=0.071$), PS1 (*d* = 15.31; FDR-corrected, $P_{\text{MSR}}=0.311$), and EOAD (*d* = 16.32; FDR-corrected, $P_{\text{MSR}}=0.433$) (Fig. 4C). Together, these results show that *AQP4* hotspots and high-atrophy regions are proximal in both anatomical space and along structural network paths. This proximity suggests that *AQP4*-enriched regions are located within anatomical and network neighborhoods commonly affected by neurodegenerative disease (*72*–*76*).

### Inflammation modulates the link between AQP4 and neurodegeneration

The relationship between the glymphatic system and neurodegeneration is thought to be intertwined with neuroinflammation. Experimental work suggests that neuroinflammatory processes can perturb AQP4 perivascular polarization, slow glymphatic clearance, and promote protein aggregation (*45*, *46*). These findings raise the possibility that the brain-wide inflammatory milieu relates to where atrophy is expressed, and that inflammation-related topography may provide information about regional vulnerability that complements *AQP4* transcriptional topography. Accordingly, we test whether normative inflammation markers explain variance in disease-related atrophy beyond *AQP4* expression, thereby strengthening the alignment between *AQP4* topography and neurodegenerative patterns.

To capture spatial variation in neuroinflammatory tone, we use normative in vivo PET maps of translocator protein (TSPO) (*78*), cyclooxygenase-1 (COX-1) (*79*), and cyclooxygenase-2 (COX-2) (*80*) in healthy adults. TSPO is an outer mitochondrial membrane protein whose PET signal is widely used as an index of neuroimmune activation, often attributed to activated glial responses, but it is not strictly cell specific and can reflect contributions from multiple cellular compartments depending on context (*81*, *82*). COX-1 and COX-2 are key enzymes in prostaglandin synthesis; COX-1 is more constitutive and is linked to microglial (innate immune) signaling, whereas COX-2 is an inducible prostaglandin pathway that is prominent in neurons and up-regulated by stress and pathology (*79*, *80*). Figure S7A shows spatial associations between *AQP4* expression and inflammatory markers. Together, these tracers provide complementary molecular indices of inflammation-related signaling, allowing us to test whether inflammatory tone modulates regional vulnerability linked to AQP4.

**Fig. 5.**
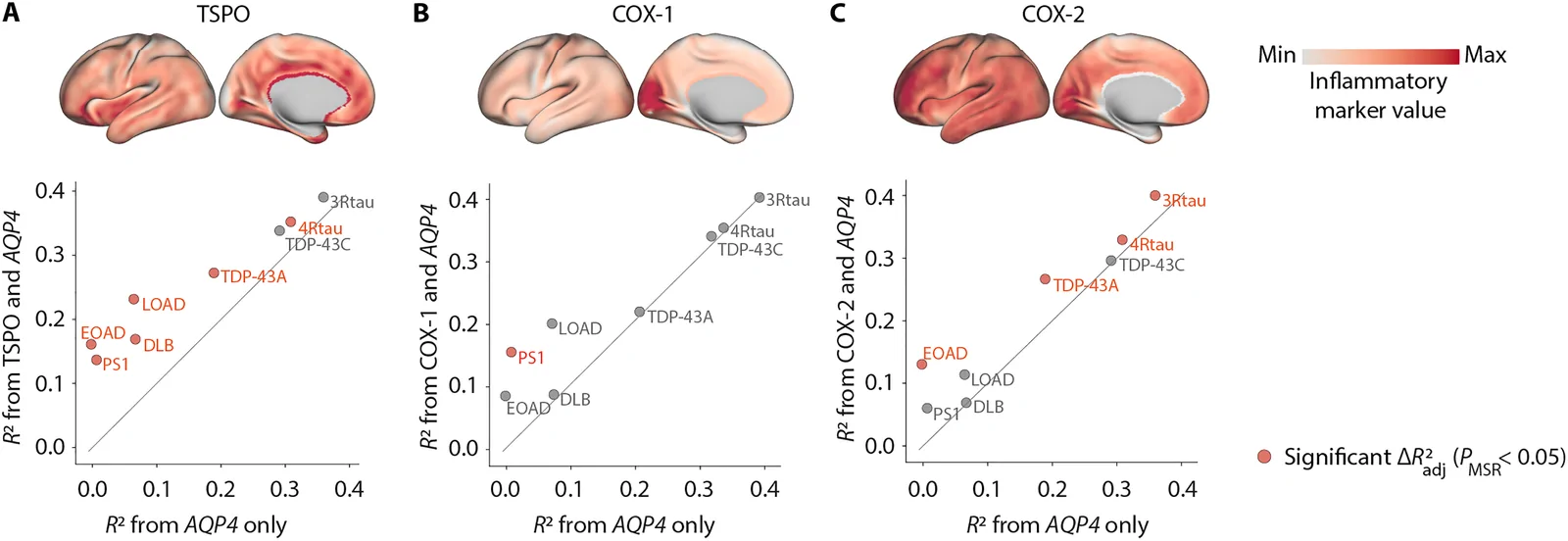
Inflammatory markers augment *AQP4*-atrophy associations. (**A** to **C**) Top: normative PET-derived maps in healthy adults for neuroinflammatory markers including TSPO (*78*), COX-1 (*79*), and COX-2 (*80*) (from left to right). Bottom: for each disease, we quantify model fit for an *AQP4*-only model (*x* axis) and for a model that additionally includes TSPO (A), COX-1 (B), or COX-2 (C) (*y* axis), using adjusted $R^{2}$ ($R_{\text{adj}}^{2}$). Each point is a disease; the identity line is shown in gray. Points above the line indicate improved fit after adding the marker. Orange points indicate significant improvements in fit relative to spatial autocorrelation–preserving null models generated with Moran spectral randomization ($P_{\text{MSR}}<0.05$) (*71*). Disease abbreviations match Fig. 3.

To test whether inflammatory markers improve the alignment between *AQP4* expression and atrophy, we first fit a simple linear model in which atrophy is predicted from *AQP4* expression alone, and quantify model fit using adjusted $R^{2}$ ($R_{\text{adj}}^{2}$). We then extend the model by adding either TSPO, COX-1, or COX-2 as an additional predictor and compute the change in model fit, $\Delta R_{\text{adj}}^{2}$. Improvements in model performance are tested against a null model in which the values of the additional predictor are randomized while preserving spatial autocorrelation using MSR (*71*). Any increases are therefore not due to the addition of a predictor or its spatial smoothness. Significant improvements are highlighted in Fig. 5 (A to C).

Figure 5 summarizes results across diseases. Across markers, most points fall above the identity line, indicating that adding an inflammatory marker generally improves the prediction of atrophy beyond *AQP4* alone. The clearest and most consistent improvements are seen for TSPO and COX-2. In particular, TSPO produces marked gains for the Alzheimer’s spectrum and Lewy body disease (EOAD, LOAD, PS1, and DLB), where the *AQP4*-only model explains relatively little variance but model fit increases substantially once TSPO is included (Fig. 5A). By contrast, COX-2 preferentially boosts fit for proteinopathies with stronger baseline coupling, most notably tauopathies and TDP-43A (Fig. 5C). In comparison, COX-1 yields smaller and less systematic changes in model fit (Fig. 5B). A complementary model, testing whether *AQP4* adds variance beyond PET markers alone, is shown in fig. S7B. Together, these findings suggest that inflammatory markers provide information about regional vulnerability that is not captured by *AQP4* expression alone, strengthening the alignment between *AQP4* transcriptional topography and disease atrophy patterns.

### AQP4 channels and susceptibility to glioma

Gliomas are primary brain tumors that invade surrounding tissue and disrupt the local brain environment. This disruption involves both tumor spread and changes in fluid balance, as gliomas are often accompanied by blood-brain barrier breakdown, abnormal vasculature, and peritumoral edema. AQP4 is relevant to these processes because it is the main astrocytic water channel and is concentrated at perivascular endfeet, where it regulates water exchange between blood vessels and brain tissue (*83*, *84*). Moreover, AQP4 is often up-regulated and redistributed in glioma, and experimental studies suggest that it contributes to tumor cell migration, invasion, and infiltration into surrounding tissue (*39*–*41*, *85*, *86*). These findings suggest that AQP4 may be associated with glioma through both tumor cell spread and altered water handling around the tumor. Motivated by these observations, we ask whether regional *AQP4* expression aligns with distributions of enhancing tumor and the surrounding peritumoral FLAIR abnormality, which includes edema as well as infiltrative or nonenhancing tumor tissue.

**Fig. 6.**
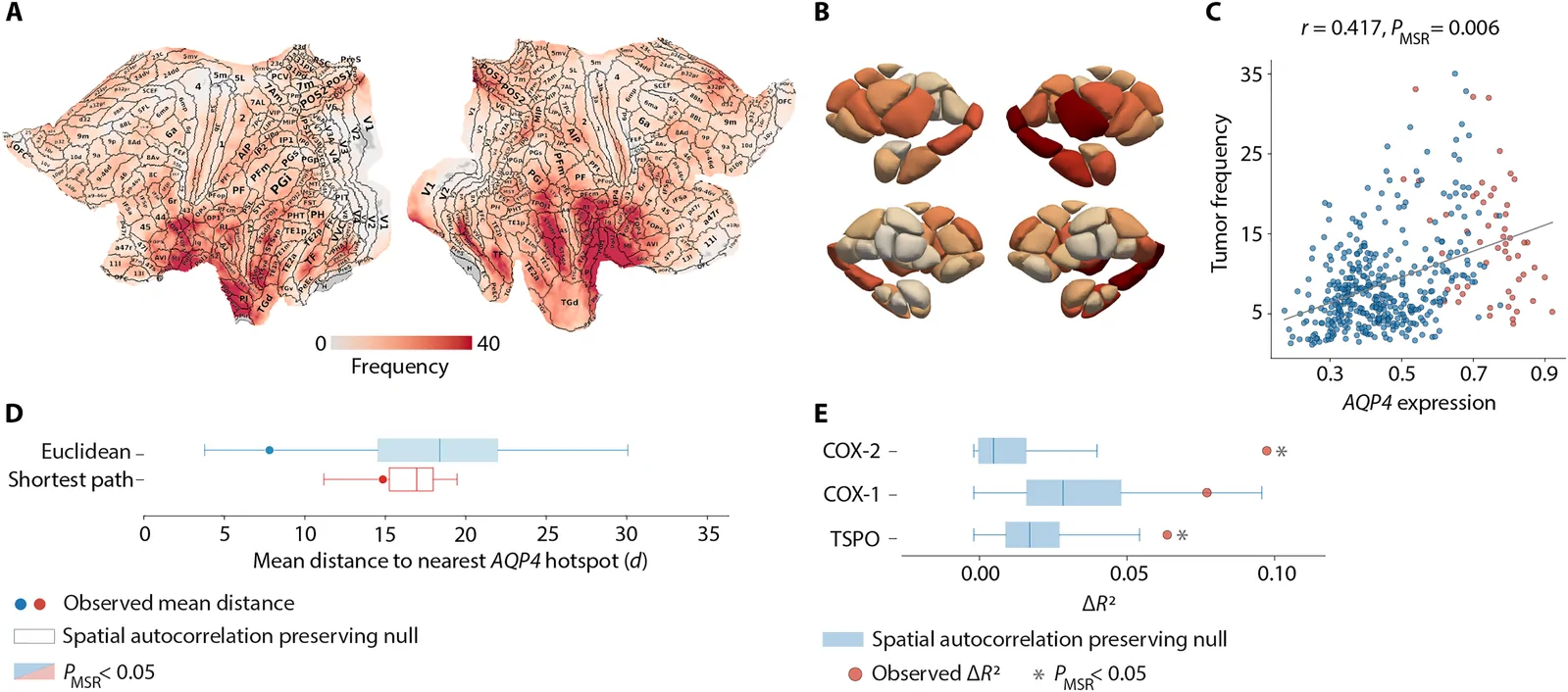
Tumor distribution aligns with *AQP4* expression. (**A** and **B**) Normative maps of tumor frequency estimated from expert tumor delineations in two preoperative glioma MRI cohorts [*N* = 501 (*87*) and *N* = 630 (*88*); see Materials and Methods]. Individual tumor masks are aggregated into a voxel-wise tumor frequency map and parcellated to the Schaefer-Melbourne atlas (*48*, *49*). (A) Cortical tumor frequency is shown on 2D flat fsLR surfaces with Glasser borders and areal abbreviations overlaid (*169*) (table S2). (B) Subcortical tumor frequency is shown in the Melbourne Subcortex S4 Atlas (*49*) (table S1 and fig. S10). (**C**) Pearson’s correlation between *AQP4* expression and tumor frequency (*r* = 0.41, $P_{\text{MSR}}=0.006$). (**D**) Proximity of high-tumor parcels (top decile of tumor frequency) to *AQP4* hotspots (top decile of *AQP4* expression), quantified as mean Euclidean distance and mean shortest path distance along the SC. Shortest path distances are computed on a group-averaged connectome derived from the HCP S1200 release (*N* = 1065; see Materials and Methods) (*77*). Observed mean distances are shown as points relative to spatial autocorrelation–preserving null distributions shown as boxplots. (**E**) Incremental variance explained ($\Delta R_{\text{adj}}^{2}$) when adding TSPO (*78*), COX-1 (*79*), and COX-2 (*80*) to an *AQP4*-only model predicting tumor frequency. Points show observed $\Delta R_{\text{adj}}^{2}$ values, and boxplots show spatial autocorrelation–preserving null distributions; asterisks denote $P_{\text{MSR}}<0.05$.

To characterize the spatial distribution of the tumor, we use expert delineations from two glioma MRI cohorts [*N* = 501; (*87*) and *N* = 630; (*88*)]. Individual tumor masks are aggregated into a voxel-wise tumor frequency map, which is then parcellated to the Schaefer-Melbourne atlas (*48*, *49*) (see Materials and Methods). Tumor frequency is not uniformly distributed across cortex and subcortex (Fig. 6, A and B). In cortex, tumor frequency is higher in ventral insular and peri-Sylvian regions and sensorimotor cortex, with additional involvement in temporal default-network territories, whereas visual cortex shows consistently low involvement (Fig. 6A). Subcortical tumor frequency is highest in medial temporal and basal ganglia territories, peaking in hippocampus, ventral putamen, posterior globus pallidus, and lateral amygdala, while it is comparatively lower across several thalamic subdivisions (Fig. 6B). We also generate a peritumoral FLAIR abnormality frequency map from the same cohorts. The peritumoral FLAIR abnormality map shows a similar spatial pattern, consistent with the expected proximity of peritumoral abnormality to the tumor itself (fig. S8, A and B).

We first ask whether tumor frequency colocalizes with regional *AQP4* expression. We find that *AQP4* expression is positively correlated with tumor frequency (Fig. 6C; *r* = 0.41, $P_{\text{MSR}}=0.006$). Moreover, high-tumor parcels show reduced Euclidean distance to *AQP4* hotspots relative to spatial autocorrelation-preserving null expectations (Fig. 6D), indicating spatial proximity between tumor-prone regions and *AQP4*-enriched territories. The peritumoral FLAIR abnormality map also shows a similar association with *AQP4* expression (fig. S8D), consistent with its close spatial coupling to the tumor itself.

We then test whether normative PET markers of neuroinflammation [TSPO (*78*), COX-1 (*79*), and COX-2 (*80*)] explain additional variance in tumor beyond *AQP4*. Using the same hierarchical regression framework as above, adding each inflammatory marker improves model fit relative to *AQP4* alone, with the largest increase observed for COX-2 (Fig. 6E; COX-2: $\Delta R_{\text{adj}}^{2}=0.097$, $P_{\text{MSR}}=0.001$; COX-1: $\Delta R_{\text{adj}}^{2}=0.077$, $P_{\text{MSR}}=0.052$; TSPO: $\Delta R_{\text{adj}}^{2}=0.063$, $P_{\text{MSR}}=0.005$). We also observe a similar pattern for the peritumoral FLAIR abnormality map (fig. S8E). Together, these results suggest that *AQP4* expression colocalizes with glioma distribution and is additionally associated with regional inflammatory tone.

### Specificity of AQP4 associations

Aquaporin-mediated water (and solute) transport in the brain is distributed across multiple isoforms. Among these, AQP1, AQP4, and AQP9 have the most consistent evidence for their roles in brain physiology and disease (*89*–*92*). AQP1 is highly expressed in the choroid plexus epithelium and has been implicated in facilitating transepithelial water permeability and CSF secretion (*93*). In contrast, AQP4 is the predominant aquaporin in the brain parenchyma, enriched in astrocytes and polarized to perivascular endfeet and the glia limitans, positioning it at key CSF-ISF interfaces implicated in perivascular exchange and clearance (*19*, *21*). AQP9, by comparison, is an aquaglyceroporin expressed in subsets of astrocytes and other neural cell types (including periventricular populations), with permeability to glycerol and other small solutes, linking it more directly to metabolic substrate transport than to purely water-selective flux (*94*, *95*). Motivated by these distinct physiological roles—and the central role of AQP4 in waste clearance and water homeostasis—we apply the same AHBA-based mapping and whole-brain association framework to AQP1 and AQP9 as within-family comparators alongside AQP4.

**Fig. 7.**
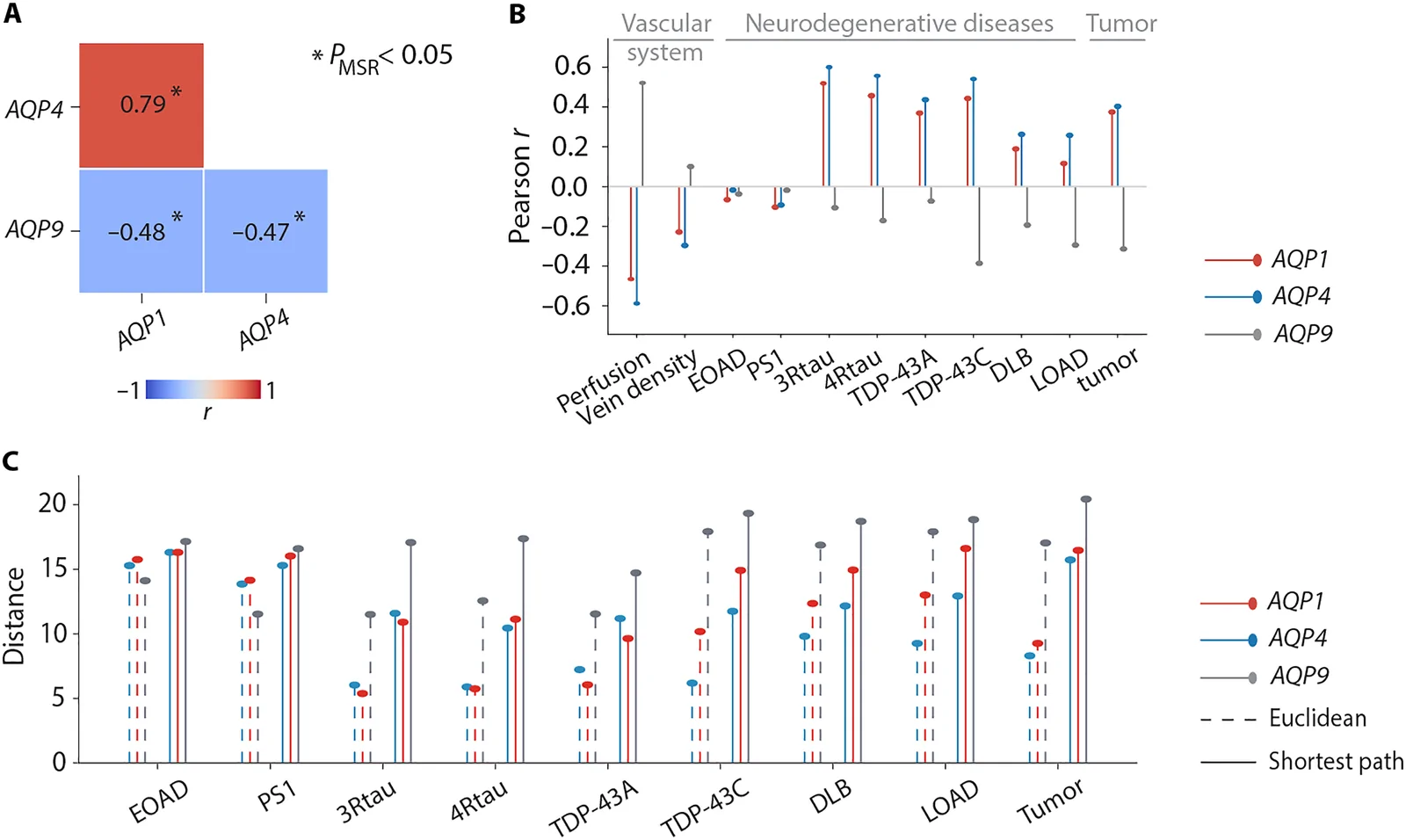
Extending to other aquaporins. (**A**) Pearson correlations among AHBA-derived expression maps of *AQP1*, *AQP4*, and *AQP9* (*2*). (**B**) Correlations between each aquaporin expression map and the phenotypes studied here: vascular measures [blood perfusion PC1 (*58*); VENAT vein density (*62*)], neurodegenerative atrophy patterns (EOAD, PS1, 3Rtau, 4Rtau, TDP-43A, TDP-43C, DLB, and LOAD) (*68*), and tumor frequency (*87*, *88*). (**C**) Mean distance from disease vulnerable parcels (top decile of atrophy or tumor) to the nearest aquaporin hotspot (top decile of expression), computed using Euclidean distance (dashed) or SC shortest path distance (solid). See Fig. 4 (A and B) for a schematic of the proximity analysis. Gene maps are processed with the abagen workflow (*47*) and parcellated to the Schaefer-Melbourne atlas (see Materials and Methods) (*48*, *49*).

First, we ask how *AQP4* covaries with *AQP1* and *AQP9* across parcels. We find that *AQP1* and *AQP4* show very similar spatial patterns (r=0.79, $P_{\text{MSR}}=1.0\times 10^{-4}$), whereas *AQP9* shows an inverse spatial alignment with both *AQP1* (r=−0.48, $P_{\text{MSR}}=0.016$) and *AQP4* (r=−0.47, $P_{\text{MSR}}=2.4\times 10^{-3}$) (Fig. 7A). We further investigate the spatial association between each aquaporin expression and the phenotypes examined in this study, including vascular measures, dementia atrophy patterns, and tumor frequency. Across phenotypes, *AQP1* shows a similar profile to *AQP4*; both show negative correlations with vascular measures and positive correlations with neurodegenerative atrophy patterns and with tumor frequency. In contrast, *AQP9* shows opposite-sign correlations (Fig. 7B).

We next ask whether vulnerable territories are preferentially located near aquaporin-enriched regions using the same hotspot proximity framework as for *AQP4*. For each gene, hotspots are defined as the top decile of expression, and we quantify the mean distance from high-atrophy (or high-tumor) parcels to the nearest hotspot using both Euclidean distance and SC shortest path distance. Figure 7C shows that vulnerable territories are generally closer to *AQP4* and *AQP1* hotspots than to *AQP9* hotspots. Together, these results suggest that the spatial coupling observed for *AQP4* is not shared uniformly across the aquaporin family.

To further assess whether the observed associations reflect *AQP4*-related topography rather than broader astrocytic, vascular, or transcriptomic gradients, we extend the specificity analysis beyond the aquaporin family. We compare *AQP4* with astrocyte-related markers (*ALDH1L1*, *GFAP*, and *SLC1A2*) (*96*–*99*), vascular and perivascular markers representing complementary components of the neurovascular unit (*ACTA2*, *PDGFRB*, *SLC2A1*, *PECAM1*, and *CLDN5*) (*100*–*103*), and the first three principal components of AHBA gene expression (*3*, *104*). *AQP4* shows positive correlations with *GFAP, PECAM1, CLDN5*, and gene expression PC1 and PC3, but its phenotype-association profile is not reproduced uniformly by these maps (fig. S9A). *GFAP, PECAM1*, and gene expression PC3 show partial similarity to *AQP4*, whereas the remaining control maps show weaker, less consistent, or more phenotype-specific associations (fig. S9B).

## DISCUSSION

In this study, we provide a whole-brain account of glymphatic organization using *AQP4* expression as a molecular anchor. We first map *AQP4* expression across cortex and subcortex using AHBA microarray data. We find the highest expression in periventricular and ventral subcortical regions, and in limbic and transmodal cortices. We next relate this spatial axis to vascular organization, neurodegenerative vulnerability, neuroinflammation, and glioma. First, *AQP4* expression is anticorrelated with normative perfusion and with a vein density atlas, suggesting that *AQP4*-enriched territories are not simply a reflection of macroscale vascular supply or large-vessel anatomy. However, high-WMH regions are close to *AQP4* hotspots, relating *AQP4* distribution to areas commonly vulnerable to CSVD. Second, *AQP4* expression colocalizes with atrophy patterns across several pathologically confirmed dementias, most strongly in tau and TDP-43 proteinopathies, and these associations often strengthen when inflammatory markers are incorporated. Third, tumor frequency aligns with *AQP4* expression, and inflammatory markers account for additional variance beyond *AQP4*. Last, comparisons within the aquaporin family suggest that these effects are not shared uniformly across isoforms. *AQP4* (and, to a lesser extent, *AQP1*) shows consistent coupling to the phenotypes examined here, whereas *AQP9* follows a distinct spatial profile. Together, our findings place *AQP4* at the intersection of perivascular fluid exchange, vascular physiology, inflammation, and vulnerability to neurodegeneration and glioma.

A central point of interpretation is that *AQP4* expression, localization, and function represent related but distinct biological levels. The AHBA-derived map used here captures regional variation in *AQP4* transcript abundance, but it does not directly measure AQP4 protein abundance, membrane trafficking, perivascular polarization, isoform composition, orthogonal array assembly, or glymphatic flux. This distinction is important because AQP4-mediated water transport depends strongly on the enrichment of AQP4 at perivascular astrocyte endfeet, the relative abundance of AQP4 isoforms, and the assembly of AQP4 into orthogonal arrays of particles in the astrocyte membrane (*57*). Experimental work shows that AQP4 can be rapidly relocalized to the cell surface in response to changes in tonicity through calcium-, calmodulin-, and protein kinase A–dependent mechanisms, indicating that functional water transport can be regulated without necessarily requiring changes in transcript abundance (*105*). Similarly, mild hypothermia increases AQP4 surface localization in human astrocytes despite no detectable change in total AQP4 protein (*106*). Therefore, we interpret the present map as a macroscale *AQP4* transcriptional topography related to perivascular water-exchange biology, not as a direct readout of glymphatic activity or clearance capacity.

AQP4 is enriched in astrocytes and shows strong subcellular polarization to perivascular endfeet and the glia limitans, placing it at major CSF-ISF interfaces implicated in perivascular exchange (*23*). In this context, the high *AQP4* expression we observe in periventricular and ventral subcortical territories—including basal ganglia and amygdalar regions—is consistent with the broader view that CSF-ISF exchange involves both perivascular routes and interfaces adjacent to CSF reservoirs, including ventricular surfaces (*57*, *107*). In cortex, higher expression in limbic and transmodal areas and lower expression in primary sensory regions suggests that *AQP4* expression follows large-scale gradients of cortical organization. In parallel, the diffusion tensor imaging analysis along the perivascular space (DTI-ALPS) index shows a clear large-scale topography, with developmental changes following a posterior-anterior axis and the strongest age-related increases moving toward anterior regions (*108*). More broadly, emerging work shows that vascular and perivascular cell properties, including astrocytic and endothelial specializations, vary systematically across brain regions (*109*). Together, these observations support the idea that *AQP4* expression is spatially heterogeneous, while direct regional differences in glymphatic function or fluid-handling capacity remain to be tested with functional measures.

Glymphatic transport is anatomically embedded in the cerebrovascular tree. Perivascular spaces surround penetrating vessels, and CSF-ISF exchange along these pathways is modulated by multiple physiological drivers, including arterial pulsatility, respiration-linked pressure changes, and slow vasomotion (*110*–*114*). Despite this coupling, we find that *AQP4* expression is anticorrelated with cerebral perfusion and with a vein density atlas. A parsimonious interpretation is that perfusion and vein density mainly reflect macroscale blood supply and large-vessel anatomy, whereas perivascular exchange depends more on perivascular spaces around penetrating small vessels and their local properties, including their geometry and the polarization of astrocytic endfeet that regulate water transport (*14*, *57*, *115*–*117*). We therefore next examine WMH, a major imaging marker of CSVD, as a complementary vascular phenotype. WMH are commonly interpreted as reflecting small vessel pathology, impaired fluid drainage, blood-brain barrier dysfunction, and white matter injury, making them relevant to perivascular clearance pathways. Prior work has linked glymphatic dysfunction and enlarged perivascular spaces to CSVD features, including WMH burden. We find that regions with high WMH burden lie close to *AQP4*-enriched regions, supporting a spatial link between *AQP4* topography and small vessel–related white matter vulnerability (*32*, *33*, *63*).

A major motivation for studying *AQP4* transcriptional topography is the long-standing hypothesis that regional neurodegenerative vulnerability is shaped, in part, by how efficiently interstitial solutes can be cleared along glymphatic outflow routes. Experimental work suggests that impaired perivascular exchange, AQP4 dysfunction, or loss of perivascular AQP4 localization can reduce amyloid-β clearance and exacerbate amyloid pathology (*18*, *118*, *119*). Parallel work supports a role for glymphatic pathways in tau handling, with impaired glymphatic function associated with reduced tau clearance and greater tau accumulation in disease models (*36*, *38*, *67*, *120*). Human studies further suggest that glymphatic dysfunction varies across individuals and brain regions, and that reactive astrogliosis and loss of AQP4 polarization contribute to impaired perivascular exchange in aging and disease (*34*, *37*, *57*, *121*). Our findings complement these studies by showing that *AQP4* expression spatially aligns with atrophy patterns across pathologically confirmed dementias, most strongly for tau and TDP-43 proteinopathies. This pattern is consistent with experimental evidence linking AQP4-dependent glymphatic clearance to extracellular tau handling. The link with TDP-43 is less direct, but emerging work in TDP-43–related neurodegeneration suggests early glymphatic dysfunction and perivascular TDP-43 pathology involving astrocytic endfeet and AQP4 loss (*122*, *123*). The association with DLB is also consistent with emerging evidence that glymphatic function and AQP4 localization influence α-synucleinopathy clearance, propagation, and seeding in α-synucleinopathy models (*124*, *125*). By contrast, the weak associations for PS1 mutation carriers and EOAD may reflect a different upstream biology. These conditions are more closely linked to amyloid pathway abnormalities, including altered amyloid precursor protein processing and amyloid-β generation, although EOAD is heterogeneous and not always mutation driven. LOAD, in contrast, has been linked more strongly to impaired amyloid-β clearance, together with vascular, inflammatory, and age-related factors (*126*–*128*). These spatial associations are compatible with at least two nonexclusive explanations. First, regions with higher *AQP4* expression may be more dependent on intact AQP4-mediated perivascular transport and therefore more vulnerable when AQP4 localization or function is disrupted. Second, *AQP4*-enriched regions may mark perivascular territories where interstitial solutes and proteins are more likely to accumulate over time, increasing susceptibility to atrophy. These explanations remain hypothetical, and the present findings should be interpreted as spatial associations rather than evidence that *AQP4* in enriched regions causally drives neurodegeneration.

Beyond map-to-map correlations, we test whether high-atrophy territories tend to lie near *AQP4*-enriched regions. We find that, for most diseases, high-atrophy regions are close to *AQP4* hotspots, both in Euclidean space and along shortest paths along the SC. This suggests that atrophy is proximal to *AQP4*-rich regions, not only in anatomical space but also in network space. More broadly, this pattern fits network-based models of neurodegeneration, where local susceptibility interacts with connectome topology and network architecture shapes the spatial patterning of disease effects (*72*–*76*). In this view, *AQP4*-rich regions may mark territories where pathology is initiated, while connections can provide routes through which vulnerability can propagate to nearby regions in network space. However, proximity does not imply causality. *AQP4* may also covary with other macroscale axes such as cytoarchitecture, myelination, or cortical hierarchy (*3*, *5*, *129*). Future work should test whether *AQP4* explains additional variance in disease topography beyond these established gradients.

In addition, our study finds that neuroinflammatory topography refines the association between *AQP4* and neurodegeneration. Across diseases, adding normative PET markers of inflammatory tone (TSPO, COX-1, and COX-2) typically improves the explanatory power of *AQP4* for atrophy, with the most consistent gains for TSPO and COX-2. This fits a view in which perivascular transport, vascular permeability, and inflammatory signaling are linked. Inflammatory activation can shift astrocyte state, disrupt endfoot organization, and reshape perivascular spaces, which can impair solute clearance. Conversely, reduced clearance can prolong exposure to extracellular debris and misfolded proteins, which can sustain neuroimmune activation (*20*, *45*, *46*, *115*). The marker-specific patterns we observe may also reflect differences in what these tracers capture. TSPO PET is widely used to index neuroimmune activation, but it is not cell specific and can reflect multiple cellular sources depending on context (*81*, *82*, *130*). COX-1 is often treated as a more constitutive prostaglandin pathway with strong myeloid/microglial contributions, whereas COX-2 is prominent in neurons and is inducible by activity and stress (*131*–*133*).

Glioma provides a complementary phenotype linking *AQP4* expression to tumor distribution and the surrounding peritumoral environment. AQP4 has been implicated in glioma through altered expression in glioblastoma, redistribution in peritumoral tissue, and possible roles in tumor cell migration and invasion (*39*–*41*, *85*, *86*). Consistent with this literature, we find higher tumor frequency in regions with higher *AQP4* expression. Because AQP4 is the main astrocytic water channel and is relevant to water handling at vessel–brain interfaces, we also examined the surrounding peritumoral FLAIR abnormality (*44*, *134*). We observe a similar pattern for this compartment. However, this result is interpreted cautiously because the FLAIR compartment is not pure edema; it may include edema as well as infiltrative or nonenhancing tumor tissue. Because this abnormality is spatially tied to tumor location, the current data cannot determine whether the association with AQP4 reflects fluid accumulation specifically or the broader distribution of glioma. Moreover, inflammatory markers account for additional variance beyond AQP4, with significant increases for COX-2 and TSPO. This is consistent with evidence that inflammatory pathways contribute to glioma progression and the tumor microenvironment. COX-2 and prostaglandin signaling have been linked to glioblastoma proliferation, invasion, immunosuppression, and poor prognosis (*135*–*137*), while TSPO PET has been used to image inflammation and immune features within the glioma microenvironment (*138*–*140*). Together, these findings suggest that regional *AQP4* expression is spatially associated with glioma frequency, while inflammatory markers capture additional spatial variance in this pattern.

We lastly investigate whether these vulnerability associations are specific to *AQP4* by repeating the analyses for related aquaporins, *AQP1* and *AQP9*. *AQP1* is enriched in the choroid plexus epithelium and is commonly linked to transepithelial water movement and CSF production (*93*, *141*), whereas AQP4 is the predominant parenchymal aquaporin, enriched in astrocytes and polarized to perivascular endfeet and the glia limitans, where it supports water exchange across vessel-brain interfaces (*14*, *19*, *21*). We find that *AQP1* largely tracks *AQP4* across phenotypes, whereas AQP9, an aquaglyceroporin with closer ties to solute and metabolic substrate transport, shows an opposite-sign association profile across phenotypes (*94*, *142*, *143*). The spatial similarity between *AQP1* and *AQP4* may reflect partially overlapping large-scale fluid-related territories rather than identical biological roles. AQP1 is more related to CSF production and epithelial water transport, whereas AQP4 is more closely related to parenchymal and perivascular water handling. Thus, the observed vulnerability associations are not shared uniformly across aquaporins and should be interpreted in light of their distinct cellular compartments and transport roles. Additional comparisons with astrocyte-related genes, vascular and perivascular gene markers, and the first three principal components of gene expression suggest that the associations between *AQP4* and phenotype are not simply a reflection of broad cell type–related or transcriptomic spatial gradients (*3*, *104*).

Measuring glymphatic function in humans remains challenging, and existing in vivo methods trade off invasiveness, spatial specificity, and scalability. A common noninvasive option is the DTI-ALPS index, which estimates water diffusion along perivascular spaces from a few periventricular ROIs and is typically reported as a single subject-level measure, not a whole-brain map (*144*). More direct in vivo evidence comes from intrathecal tracer MRI (often gadobutrol), which measures regional tracer enrichment and delayed clearance; however, this technique is invasive and requires repeated scans over long time windows. Here, we use *AQP4* expression as a brain-wide molecular axis related to perivascular water exchange, following a broader strategy in which gene transcription is used to map distributed molecular systems at the macroscale (e.g., neuropeptide receptor families) (*145*, *146*). This atlas-style approach is also consistent with observations in NMOSD, where brain lesions preferentially occur in regions with high *AQP4* expression, supporting the idea that *AQP4* topography is informative for where AQP4-mediated pathology manifests in the brain (*29*). Encouragingly, we find a positive spatial correlation between *AQP4* expression and gadobutrol tracer MRI measures from Ringstad and colleagues (*55*). We also find that *AQP4* expression is positively associated with AQP4 protein abundance (*52*). This provides an additional protein-level validation that the macroscale *AQP4* expression map captures biologically meaningful regional variation, supporting *AQP4* topography as a proxy for the glymphatic system.

While our study offers promising insights, several methodological limitations should be noted. First, we use bulk microarray gene expression data from the AHBA, which, despite being the most spatially comprehensive dataset currently available, is limited in resolution and sample size (*147*, *148*). Although we validate the *AQP4* spatial pattern against AHBA RNA-seq and the HPA, the extent to which it captures interindividual variability remains uncertain. Second, glymphatic function depends on perivascular AQP4 polarization and channel organization (including isoform balance and formation of orthogonal arrays), which are not measured here (*21*). Third, our analyses use maps from different cohorts and modalities (ASL, QSM, PET, VBM, and tumor MRI), so we cannot infer within-subject associations among perfusion, inflammation, AQP4, and vulnerability. Fourth, the SC used in this study is reconstructed from diffusion-weighted imaging, which is prone to false positives and false negatives (*149*, *150*). Although we minimize this limitation by using modern multishell diffusion data and state-of-the-art tractography, the inherent challenges of tractography remain. Last, disease maps are cross-sectional summaries of atrophy and do not capture longitudinal progression, which limits inference about propagation mechanisms.

Looking ahead, we hope to get all these measures (glymphatic function, perfusion, inflammation, and vulnerability) in the same individuals. In parallel, postmortem histology and single-cell measurements could clarify how regional *AQP4* transcription relates to protein abundance, isoform composition, and perivascular polarization across the life span and in disease. Furthermore, longitudinal neurodegenerative cohorts will be valuable to test whether alignment to AQP4 predicts future spread, whether inflammatory markers identify periods of accelerated vulnerability, and whether interventions that restore AQP4 polarization or modulate inflammation preferentially benefit AQP4-enriched territories. We make our code and data openly available to facilitate further research and validation of our results.

## MATERIALS AND METHODS

### Gene expression data

Gene expression data for *AQP1, AQP4, AQP9, MLC1, GFAP, ALDH1L1, SLC1A2, ACTA2, PDGFRB, SLC2A1, CLDN5,* and *PECAM1* were obtained from six postmortem brains [one female, ages 24 to 57 years, mean ± SD = 42.5 ± 13.4, three of Caucasian ethnicity, two African American, one Hispanic, (*2*)] using the abagen toolbox [v0.1.4; https://github.com/rmarkello/abagen (*47*)]. Detailed information about data acquisition can be found in (*2*). Preprocessing of gene expression data followed the procedures described in (*151*). Microarray probes for all individuals were fetched from https://human.brain-map.org/ and re-annotated to match the gene symbol ID and name from the latest version of NCBI (*152*), available at https://ftp.ncbi.nih.gov/gene/DATA/GENE_INFO/Mammalia/. Probes not matched to a valid ID were discarded. The remaining probes underwent intensity-based filtering, where probes with intensity less than background noise in less than 20% of samples across individuals were excluded. For genes represented by multiple probes, the probe with the highest average Pearson correlation to the RNA-seq data from two individuals in the dataset was selected. This ensured biological plausibility of the microarray measurements wherever possible (*153*). Tissue sampling locations from the original T1w scans of individuals were nonlinearly registered to MNI152 (1 mm) space as in https://github.com/chrisgorgo/alleninf. Probe samples were then assigned to brain regions in a standard atlas consisting of the Schaefer 400 cortical (*48*) and the Melbourne Subcortical S4 (*49*), thresholded at 0.5 probability. Samples were then assigned to a brain region with MNI coordinates within a 2-mm vicinity. To reduce the potential for misassignment, sample-to-region matching was constrained by hemisphere and gross structural divisions (i.e., cortex, subcortex/brainstem, and cerebellum), such that, e.g., a sample in the left cortex could only be assigned to an atlas voxel in the ipsilateral side. If a brain region was not assigned a sample based on that procedure, voxels in that region were mapped to the nearest tissue sample from the same individual to produce a dense, interpolated expression map. The mean of these expression values was taken across all voxels in the region, weighted by the distance between each voxel and the sample mapped to it, to obtain an estimate of the parcellated expression values for the missing region. Intersubject variation was addressed by normalizing sample expression values across genes using a robust sigmoid function as in (*13*). Normalized expression values were then rescaled from 0 to 1 by min-max normalization. Gene expression values were subsequently normalized across regions using an identical procedure. All available tissue samples were used for normalization, whether or not they were assigned to a brain region. Tissue samples not matched to a brain region were discarded after normalization. Samples assigned to the same brain region were averaged separately for each individual, which resulted in six expression matrices, one for each individual, with rows corresponding to brain regions and columns corresponding to genes. From these initial expression matrices, we retained genes with a differential stability value greater than 0.1 (*3*). All matrices were lastly mean averaged across individuals, resulting in a single matrix representative of the expression level of a particular gene in a given region.

For visualization only, a dense *AQP4* expression was generated in MNI152-NonLinear2009cAsym space using nearest-neighbor interpolation with the abagen workflow. This volumetric map was then projected to fsLR surface space for cortical display. All statistical analyses were performed on region-wise expression values as described above.

### Neurodegenerative disease atrophy data

Data for eight neurodegenerative diseases were taken from the VBM maps released by Harper *et al.* (*68*) (available via NeuroVault: https://neurovault.org/collections/ADHMHOPN/). The dataset comprises T1-weighted MRI from 186 participants with a clinical dementia diagnosis and histopathological confirmation of the underlying disease (postmortem or biopsy), as well as 73 healthy controls. Condition-level maps reflect averages within each diagnostic group: Alzheimer’s disease (*N* = 107; early-onset *N* = 68, late-onset *N* = 29, PSEN1 mutation carriers *N* = 10), DLB (*N* = 25), 3Rtau (*N* = 11), 4Rtau (*N* = 17), TDP-43A (*N* = 12), and TDP-43C (*N* = 14). Scans were acquired across multiple sites and vendors (Philips, GE, Siemens) with heterogeneous protocols; field strengths included 1.0 T (*N* = 15), 1.5 T (*N* = 201), and 3 T (*N* = 43). Neuropathological assessment followed the standard criteria used at the time of evaluation at one of four centers (Queen Square Brain Bank, King’s College Hospital, VU Medical Center Amsterdam, and the Institute for Ageing and Health, Newcastle) (*68*).

Regional tissue loss was quantified using VBM on T1-weighted images and reported as voxelwise *t* statistics. The resulting atrophy maps were adjusted for age, sex, total intracranial volume, scanner field strength, and acquisition site. The volumetric atrophy maps were parcellated to the Schaefer 400 cortical atlas (*48*) combined with the Melbourne subcortical S4 atlas (*49*). Ethics approval for the retrospective study is described in Harper *et al.* (*68*).

### Structural connectivity data

The template diffusion-weighted MRI (dMRI) connectome used to compute structural shortest paths was derived from 1065 participants in the HCP S1200 release. dMRI data were acquired with a spin-echo echo-planar imaging (EPI) sequence (TR = 5520 ms, TE = 89.5 ms, flip angle = 78°, 1.25 mm isotropic voxels) using *b* values of 1000, 2000, and 3000 s/mm^2^. Three diffusion runs with different gradient direction sets were collected (90 directions total). Full acquisition details are reported elsewhere (*77*, *154*). Preprocessing followed the HCP Minimal Preprocessing Pipelines (*155*), including intensity normalization, susceptibility distortion correction (topup), eddy-current and head-motion correction (eddy), and gradient nonlinearity correction. We downloaded bedpostX-derived fiber orientation density function estimates from the HCP S1200 release and performed probabilistic tractography with probtrackx2 to estimate inter-regional structural connectivity [QuNex v0.98.0, dwi_probtrackx_dense_gpu; (*156*)] using the Schaefer 400 cortical atlas (*48*) combined with the Melbourne subcortical S4 atlas (*49*).

### Shortest path retrieval

SC was encoded as an undirected weighted graph G≡{V,W} composed of nodes $V=\left\{v_{1},v_{2},\ldots ,v_{n}\right\}$ and a matrix of fiber density values $W=\left[w_{\mathrm{ij}}\right]$, valued on the interval [0,1]. To recover shortest paths, we first define a topological distance measure. The weighted adjacency matrix was transformed from connection weights to connection lengths using the transform L=1/W, such that connections with greater weights are mapped to shorter lengths (*157*). Note that other transformations are also possible, including L=−log(W). Weighted shortest paths were recovered using the Floyd-Warshall algorithm [(*158*–*160*); *BrainConn* Python Toolbox]. Note that in many types of networks, there may exist multiple shortest paths between two nodes (edge-disjoint or not); in our network, this was not the case, as we computed weighted shortest paths yielding unique paths between all source-target pairs.

### Spatial autocorrelation–preserving null models

Many of the maps used in this study (*AQP4* expression, perfusion, venous density, inflammation biomarkers, and disease atrophy) exhibit strong spatial autocorrelation. Standard parametric tests, therefore, overestimate the effective degrees of freedom (*69*, *70*). To obtain more appropriate significance estimates, we assessed all spatial associations against null models that preserve the empirical spatial autocorrelation using MSR, as implemented in the BrainSpace toolbox (https://brainspace.readthedocs.io/en/latest/) (*161*).

For each analysis, we generated 10,000 surrogate maps with matched spatial smoothness. MSR first quantifies spatial autocorrelation in the empirical map using Moran’s *I*, defined with a spatial weight matrix based on the inverse Euclidean distance between parcel centroids in MNI space. The corresponding spatial eigenvectors (Moran eigenvector maps) provide an orthogonal basis that captures the spatial dependence structure of the data. Surrogate maps are then obtained by sampling random coefficients in this eigenvector basis and recombining them such that the resulting maps retain the same overall spatial autocorrelation as the empirical map, while otherwise being random (*69*, *161*).

For pairwise correlations (e.g., *AQP* versus perfusion, venous density, or atrophy), we held one map fixed and applied MSR to the other map to obtain a null distribution of correlation coefficients. For the proximity analyses, we generated MSR surrogates of the disease or tumor map, redefined high atrophy or high tumor for each surrogate, and recomputed Euclidean and shortest path distances. Unless otherwise noted, *P*_MSR_ values are one sided in the direction of the observed effect.

### Blood perfusion data

Cerebral blood perfusion data were obtained from the HCP Lifespan studies (HCP-Development and HCP-Aging), comprising 1305 healthy participants spanning childhood to late adulthood (*59*–*61*). Perfusion is estimated using pseudo-continuous arterial spin labeling (pCASL) MRI acquired on a 3-T Siemens Prisma scanner with a 32-channel head coil. The pCASL sequence used a 2D multiband EPI readout with a labeling duration of 1500 ms and five postlabeling delays (200, 700, 1200, 1700, and 2200 ms), yielding 6, 6, 6, 10, and 15 control-label pairs at each delay. Additional parameters included an isotropic voxel size of 2.5 mm and TR/TE of 3580/18.7 ms, together with two M_0_ calibration images for quantification in ml 100 g^−1^ min^−1^.

ASL data were processed with the HCP ASL pipeline as implemented in QuNex (*156*). Label, control, and calibration images were coregistered to each participant’s anatomical space, and motion, susceptibility distortion, and gradient nonlinearity effects were corrected in a single resampling operation. We then applied bias-field and banding artifact corrections and performed motion-aware label-control subtraction. Cerebral blood flow and arterial transit time were computed in oxford_asl using a multicompartment Buxton model, with partial-volume effects handled via a spatial variational Bayes procedure. Quantitative perfusion maps were subsequently projected to grayordinates (cortical surface plus subcortical voxels).

To derive a normative perfusion topography shared across participants, we summarized individual perfusion maps using principal components analysis (PCA), following prior work (*58*). Briefly, participant-level perfusion maps were *z*-scored and concatenated into a participant-by-grayordinate data matrix. This participant-wise standardization reduces global offsets in perfusion levels (e.g., differences related to biological sex) and emphasizes shared spatial patterning. PCA was then applied to this matrix, and the first principal component (PC1) was retained as the group-level “perfusion score” map, as it captures the dominant regional pattern of perfusion shared across individuals (PC1 explained 50.71% of the variance; PC2 explained 2.35%). The resulting group perfusion map was then parcellated to the Schaefer 400 cortical (*48*) and Melbourne subcortical S4 (*49*) atlases to obtain regional perfusion estimates.

### WMH data

WMH data were obtained from the Meta VCI Map Consortium, using the voxel-wise WMH probability map released by Coenen *et al.* (*64*). The dataset was designed to characterize the spatial distribution of WMH in a large memory-clinic population. It included individual participant data from 3525 participants across 11 memory-clinic cohorts, comprising 777 participants with subjective cognitive decline, 1389 participants with mild cognitive impairment, and 1359 patients with dementia. WMH segmentations were provided by the participating centers or performed centrally in Utrecht. All segmentations were registered to the MNI-152 template to allow spatial comparison across cohorts and participants. A group-level WMH probability map was then generated by calculating the voxel-wise frequency of WMH across all participants. Each voxel value therefore reflects how often that location was affected by WMH in the pooled cohort, with higher values indicating more common WMH occurrence. For proximity analyses, high-WMH regions were defined as voxels with WMH frequency values greater than or equal to 90% of the maximum value in the WMH map.

### Vein density data

Normative atlas of cerebral veins was obtained from the VENAT dataset (*62*). VENAT was built from 7-T QSM acquired at 0.6-mm isotropic resolution in healthy young adults, with five repeated scans per participant (*N* = 20; mean age 25.1 ± 2.5 years). Veins were segmented in 3D and aligned across participants using a two-step registration procedure. The atlas provides voxelwise maps of average vessel location, which we treat as a vein density (or vein probability) map, and also includes summary maps of vessel diameter (mean 0.84 ± 0.33 mm) and curvature (mean 0.11 ± 0.05 mm^−1^). In our analyses, we used the average vessel location map as the vein density phenotype. We parcellated it to the Schaefer 400 cortical atlas combined with the Melbourne subcortical S4 atlas (*48*, *49*).

### Glioma data

Glioma maps were derived from two publicly available preoperative MRI resources: the UCSF Preoperative Diffuse Glioma MRI (UCSF-PDGM) dataset and the University of Pennsylvania glioblastoma (UPenn-GBM) cohort (*87*, *88*). UCSF-PDGM includes 501 adults with histopathologically confirmed diffuse gliomas spanning World Health Organization (WHO) grades 2 to 4, imaged with a standardized preoperative 3-T MRI protocol and accompanied by multicompartment tumor segmentations (*87*). UPenn-GBM comprises 630 patients with de novo glioblastoma overall and provides multimodal MRI data together with computationally derived and manually revised tumor subregion annotations (*88*).

Both datasets include standard glioma MRI sequences, including T2-FLAIR and postcontrast T1-weighted images acquired after gadolinium administration. In UCSF-PDGM, tumor compartments were generated using automated segmentation methods developed for the BraTS challenge and were then manually corrected and approved by experienced neuroradiologists (*87*). In UPenn-GBM, tumor subregions were generated using automated deep learning segmentation methods and were manually reviewed and corrected when needed (*88*). Enhancing tumor masks were obtained from the contrast-enhancing tumor compartment, defined on postcontrast T1-weighted images. Peritumoral FLAIR abnormality masks were obtained from the surrounding T2-FLAIR hyperintense compartment. In UCSF-PDGM, this compartment includes nonenhancing tumor and associated edema, whereas in UPenn-GBM, it is described as peritumoral edematous or infiltrated tissue (*87*, *88*). We therefore refer to this label as peritumoral FLAIR abnormality rather than pure edema.

To generate group-level maps, individual enhancing tumor and peritumoral FLAIR abnormality masks were transformed to MNI152 standard space and resampled to a common voxel grid. We then computed voxel-wise frequency maps by summing binary masks across participants, such that each voxel value reflects the number of individuals whose mask included that voxel. This was done separately for enhancing tumor and peritumoral FLAIR abnormality. Last, each frequency map was parcellated to the Schaefer 400 cortical atlas (*48*) combined with the Melbourne subcortical S4 atlas (*49*), yielding regional frequency scores for each parcel in the 454-region whole-brain parcellation.

### PET markers of neuroinflammation data

PET-derived maps of neuroinflammatory markers were obtained from normative atlases in healthy adults. We downloaded TSPO and COX-1 maps from the neuromaps toolbox (https://github.com/netneurolab/neuromaps), which curates a library of brain annotations and provides standardized access and transformations across common coordinate systems (*162*). Specifically, we used a TSPO PET map derived from (*78*) (radioligand: [^11^C]PBR28) and a COX-1 PET map derived from (*79*) (radioligand: [^11^C]PS13), as distributed in the neuromaps reference dataset. Methodological details regarding tracer acquisition, quantification, and preprocessing are reported in the original studies and in the neuromaps documentation (https://netneurolab.github.io/neuromaps/listofmaps.html). Furthermore, a normative COX-2 PET map was obtained from (*80*) (radioligand: [^11^C]MC1). Volumetric PET images were parcellated to the Schaefer 400 cortical (*48*) and Melbourne subcortical S4 (*49*) atlases to obtain regional estimates.

### Intrathecal CSF tracer enrichment data

As an in vivo tracer-based assay of the glymphatic system, we used regional brain enrichment measures from a prior MRI study by Ringstad *et al.* (*55*). In that study, reference participants (REF; *N* = 8, evaluated for suspected CSF leak/idiopathic intracranial hypotension) underwent repeated T1-weighted MRI before and after intrathecal administration of the CSF tracer gadobutrol (0.5 ml; 1.0 mmol/ml; Gadovist, Bayer). MRI data were acquired on a 3-T Philips Ingenia scanner using a consistent 3D T1-weighted protocol across time points. Postcontrast T1-weighted scans were repeated during the first hour, approximately every 2 hours until late afternoon, and again the next morning (approximately 24 hours postinjection). Scans were subsequently grouped into predefined time windows, including a 24-hour interval.

Image processing was performed in FreeSurfer (v6.0) (*163*) using a longitudinal workflow with within-subject template construction and rigid alignment across sessions (*164*). Tracer-related signal was quantified for each FreeSurfer-derived segmented region by computing the median T1 intensity and normalizing it to a reference ROI placed in the posterior superior sagittal sinus, yielding normalized T1 signal units that mitigate global intensity scaling (*55*). Regional tracer enrichment was then expressed as percentage change from the precontrast baseline (*55*).

In the present work, we focus on the REF group at 24 hours because this is a standardized delayed time point in which gadobutrol has distributed broadly beyond the early peri-surface phase, providing a stable snapshot of net parenchymal enrichment suitable for spatial comparison with *AQP4* expression (*55*).

### HPA gene expression data

HPA gene expression measures were obtained from RNA sequencing (RNA-seq) of micropunch samples collected at the Human Brain Tissue Bank, Semmelweis University (Hungary), spanning 190 brain regions, areas, and subfields (*50*, *51*). RNA was extracted with the RNeasy Plus Mini Kit (Qiagen), and mRNA was enriched via ribosomal RNA depletion. RNA quality was assessed using the Experion RNA HighSens Analysis kit (Bio-Rad), and only samples passing minimum thresholds for RNA Integrity Number (RIN) and the 260/280 absorbance ratio were retained. Libraries were generated with Illumina TruSeq Stranded mRNA reagents and sequenced on an Illumina NovaSeq 6000 system (paired-end, 150 bp). Reads were aligned to GRCh37/hg19 using Ensembl gene models (v92) and quantified with Kallisto (v0.43.1) (*50*, *165*, *166*). Following quality control, expression was summarized as TPM and then further normalized using trimmed mean of *M* values in NOISeq, with batch effects adjusted using limma (*167*). Normalized TPM values were log-transformed (log_10_) and assigned to Destrieux atlas regions via region-name matching (*168*).

### Brain proteomic data

Normative maps of regional protein abundance were obtained from a multiregional proteomic survey of the postnatal human brain (*52*). This dataset leveraged label-free quantitative (LFQ) tandem mass spectrometry to characterize the human brain proteome across 16 individuals spanning a developmental range from early infancy to adulthood (4 months to 42 years). Proteomic measurements were quantified for seven major brain regions: dorsolateral prefrontal cortex, primary visual cortex, hippocampus, amygdala, mediodorsal nucleus of the thalamus, striatum, and cerebellum. To generate a group-level map of protein distribution, we extracted normalized LFQ intensities for the AQP4 protein and averaged these values across all 16 participants within each of the seven regions. To enable spatial comparison with other maps, these macro-regional abundance scores were assigned to the cortical Destrieux atlas and the subcortical Melbourne S1 atlas via region-name matching (*49*, *168*).
