# Supplementary material for: Spatial organization of AQP4 water channel expression in the human brain
Source: Sci Adv. 2026 Sep 23;12(39):eaeg8117. doi: 10.1126/sciadv.aeg8117 (PMC13600213; doi:10.1126/sciadv.aeg8117)
Supplement: Supplementary file 1 [file sciadv.aeg8117_sm.pdf]

Science Advances
AAAS

Supplementary Materials for

Spatial organization of AQP4 water channel expression in the human brain

Tahmineh Taheri *et al.*

Corresponding author: Bratislav Misic, bratislav.misic@mcgill.ca

Sci. Adv. **12**, eaeg8117 (2026)
DOI: 10.1126/sciadv.aeg8117

This PDF file includes:

Figs. S1 to S10
Tables S1 and S2

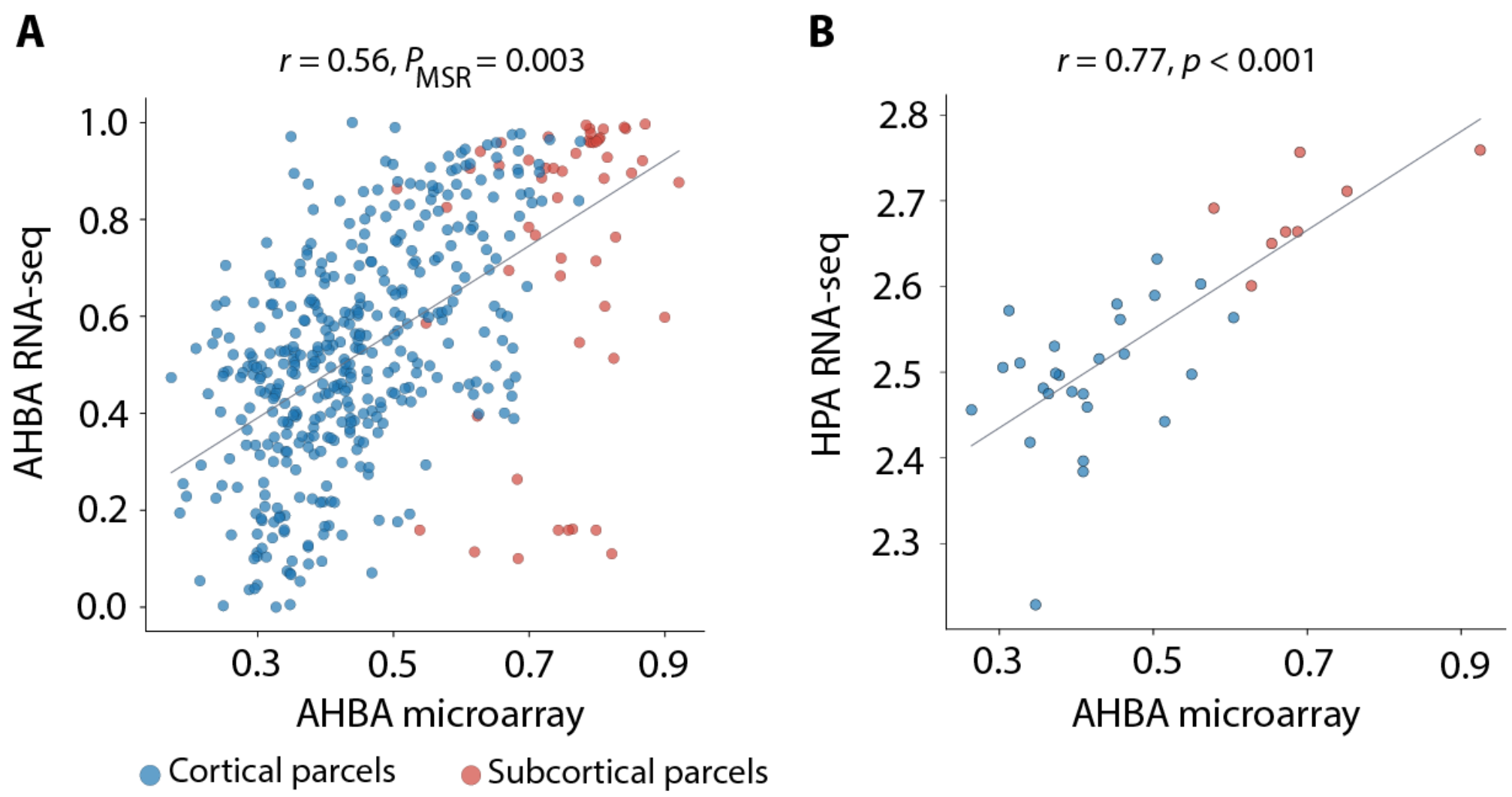

Figure S1. **Comparisons of *AQP4* expression with RNA-seq data from AHBA and the Human Protein Atlas (HPA)** | To assess the consistency of spatial patterns of *AQP4* expression in our discovery dataset (microarray measurements from the Allen Human Brain Atlas, AHBA) *(2)*, we systematically compare it with two independent RNA-seq datasets. (**A**) Parcel-wise *AQP4* expression from AHBA microarray is plotted against AHBA RNA-seq estimates across cortical and subcortical parcels (Schaefer–Melbourne parcellation *(25, 50)*; blue, cortical parcels; red, subcortical parcels). (**B**) AHBA microarray *AQP4* expression is parcellated according to the cortical Destrieux atlas *(169)* and subcortical Melbourne subcortical S1 atlas *(25)*, then matched to RNA-seq measurements from the Human Protein Atlas *(51, 52)*, yielding 26 cortical and 8 subcortical regions of interest. In both panels, the grey line shows the best-fitting linear regression, and the reported statistics give the correlation coefficient and associated p-value.

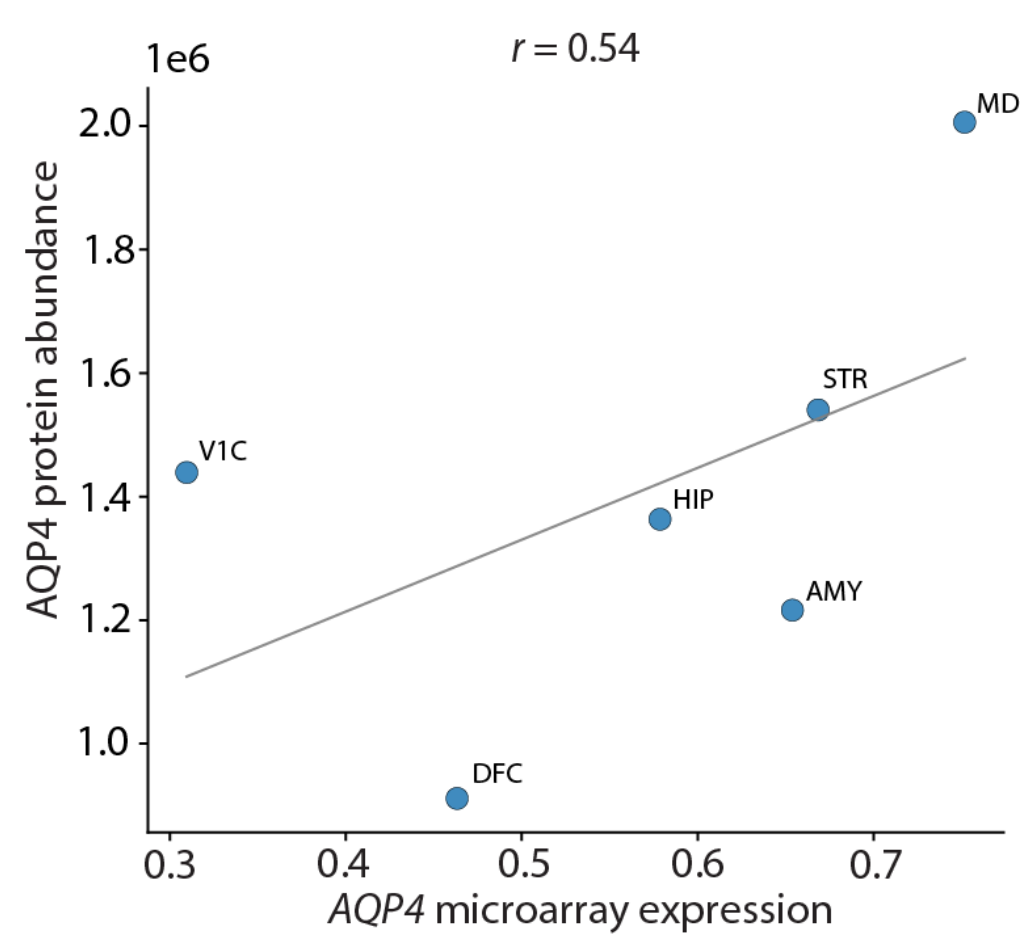

Figure S2. **Regional correspondence between *AQP4* expression and AQP4 protein abundance** | AHBA microarray *AQP4* expression *(2)* is parcellated according to the cortical Destrieux atlas *(169)* and subcortical Melbourne S1 atlas *(25)*, then matched to regional AQP4 protein abundance from an independent human brain proteomics dataset *(53)*. Matched regions included amygdala (AMY), dorsolateral prefrontal cortex (DFC), hippocampus (HIP), mediodorsal thalamus (MD), primary visual cortex (V1C), and striatum (STR). The grey line shows the least-squares fit.

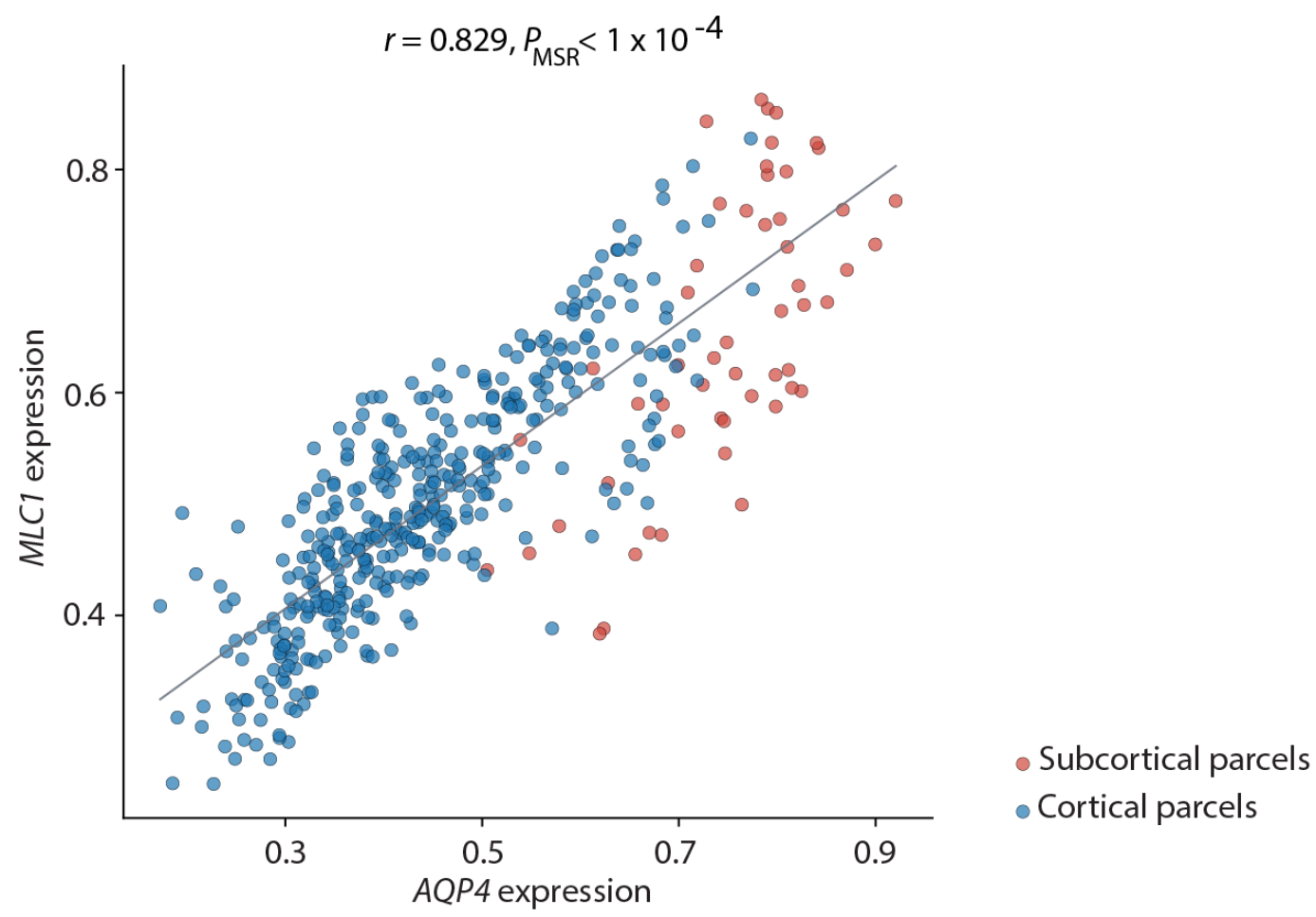

Figure S3. **Spatial association between the expression of *AQP4* and *MLC1* genes |** AHBA-derived *AQP4* expression is compared with MLC1 expression across the Schaefer–Melbourne parcellation *(2, 25, 50)*. The grey line shows the least-squares fit.

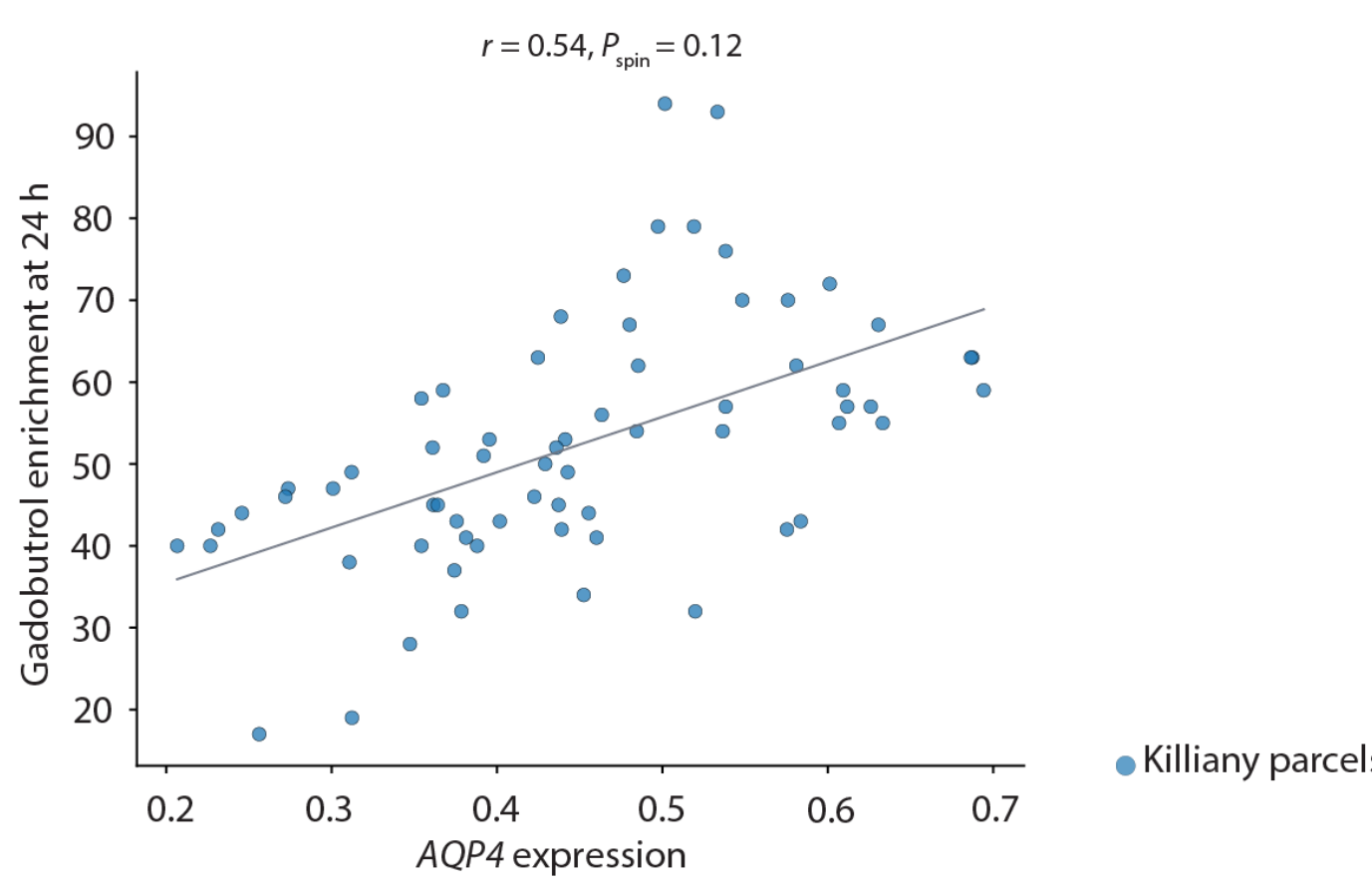

Figure S4. ***AQP4* expression correlates with delayed intrathecal CSF tracer enrichment |** Scatterplot shows parcel-wise Pearson correlation between *AQP4* expression estimated from AHBA microarray data *(2)* (x-axis) and regional enrichment of an intrathecally administered MRI tracer (gadobutrol) measured 24 hours after injection in reference participants from Ringstad et al. *(56)* (y-axis). Tracer enrichment values reflect the 24 hour percent change in normalized T1 signal relative to the pre-contrast baseline, quantified over FreeSurfer-derived anatomical regions *(164, 165, 170)*. *AQP4* values are mapped to the same FreeSurfer/Desikan–Killiany parcellation used for the tracer analysis to enable direct spatial comparison. The grey line shows the best-fitting least-squares regression.

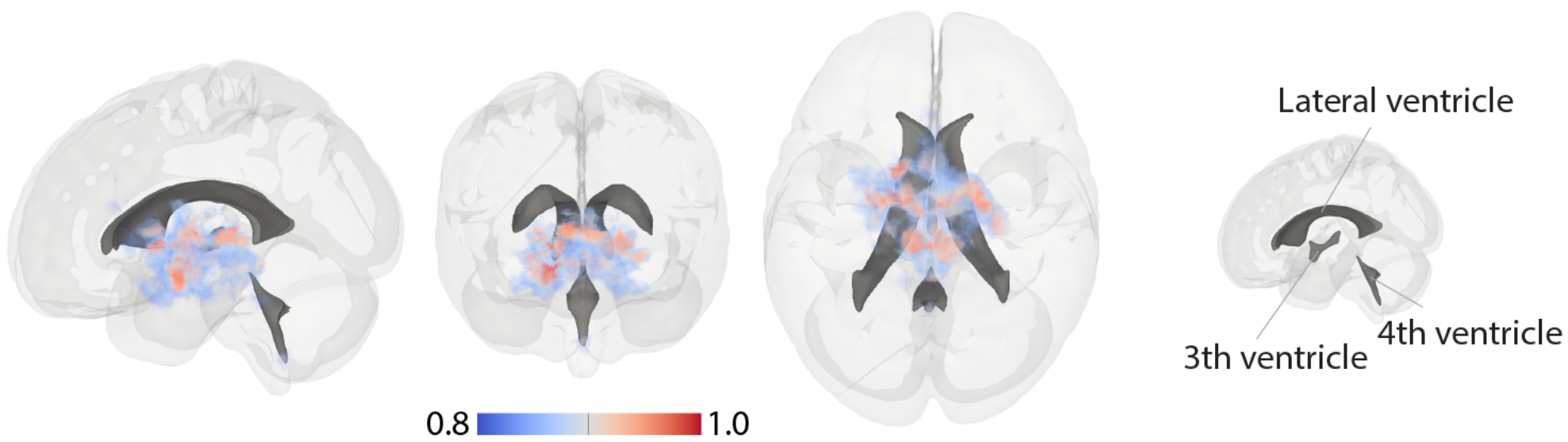

Figure S5. **Spatial distribution of high *AQP4* expression |** A dense, interpolated volumetric map of *AQP4* expression was generated from Allen Human Brain Atlas (AHBA) microarray data using the `abagen` workflow (see *Methods*) *(2, 24)* and rendered in MNI152NLin2009cAsym (1 mm) space. Only voxels with normalized expression values in the range 0.8–1.0 are shown as a volume rendering (blue–red scale). Ventricular anatomy (lateral, 3rd, and 4th ventricles; FreeSurfer aseg labels 4, 43, 14, and 15) is overlaid in grey for anatomical reference, showing that high *AQP4* expression tends to concentrate near ventricular spaces. Left-to-right: sagittal, coronal, and axial views.

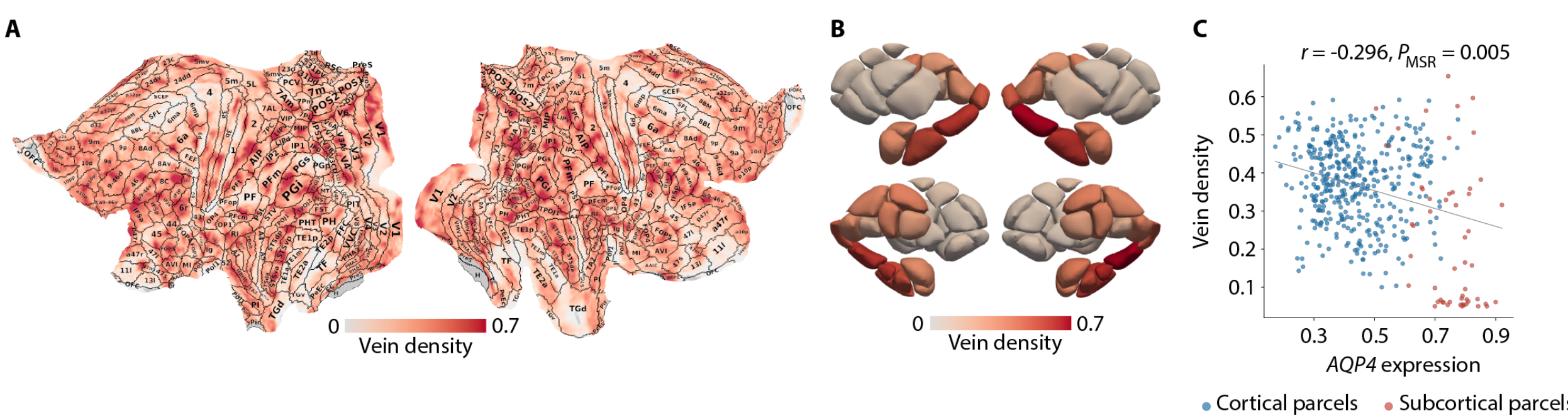

Figure S6. **Spatial association between *AQP4* and vein density |** (**A**) Cortical vein density from the VENAT atlas is shown on a 2D flat fsLR surface *(63)*. Borders and areal names from the multi-modal Glasser parcellation are overlaid on the flat surface *(26)*; the full Glasser nomenclature is provided in Table S2. (**B**) Subcortical vein density parcellated to the Melbourne subcortical S4 atlas *(25)* and shown as 3D parcel surfaces across multiple views. Subcortical parcel names and abbreviations are listed in Table S1, and labeled parcel renderings are shown in Fig. S10. (**C**) Pearson correlation between *AQP4* expression from the Allen Human Brain Atlas and vein density ($r = -0.296$, $p_{\mathrm{MSR}} = 0.005$). Each point is a parcel (blue: Schaefer 400 cortical parcels *(50)*; red: Melbourne S4 subcortical parcels *(25)*). The grey line shows the least-squares fit. p_{MSR} value is computed using Moran spectral randomization ($N_{\mathrm{MSR}} = 10\,000$) *(66)*.

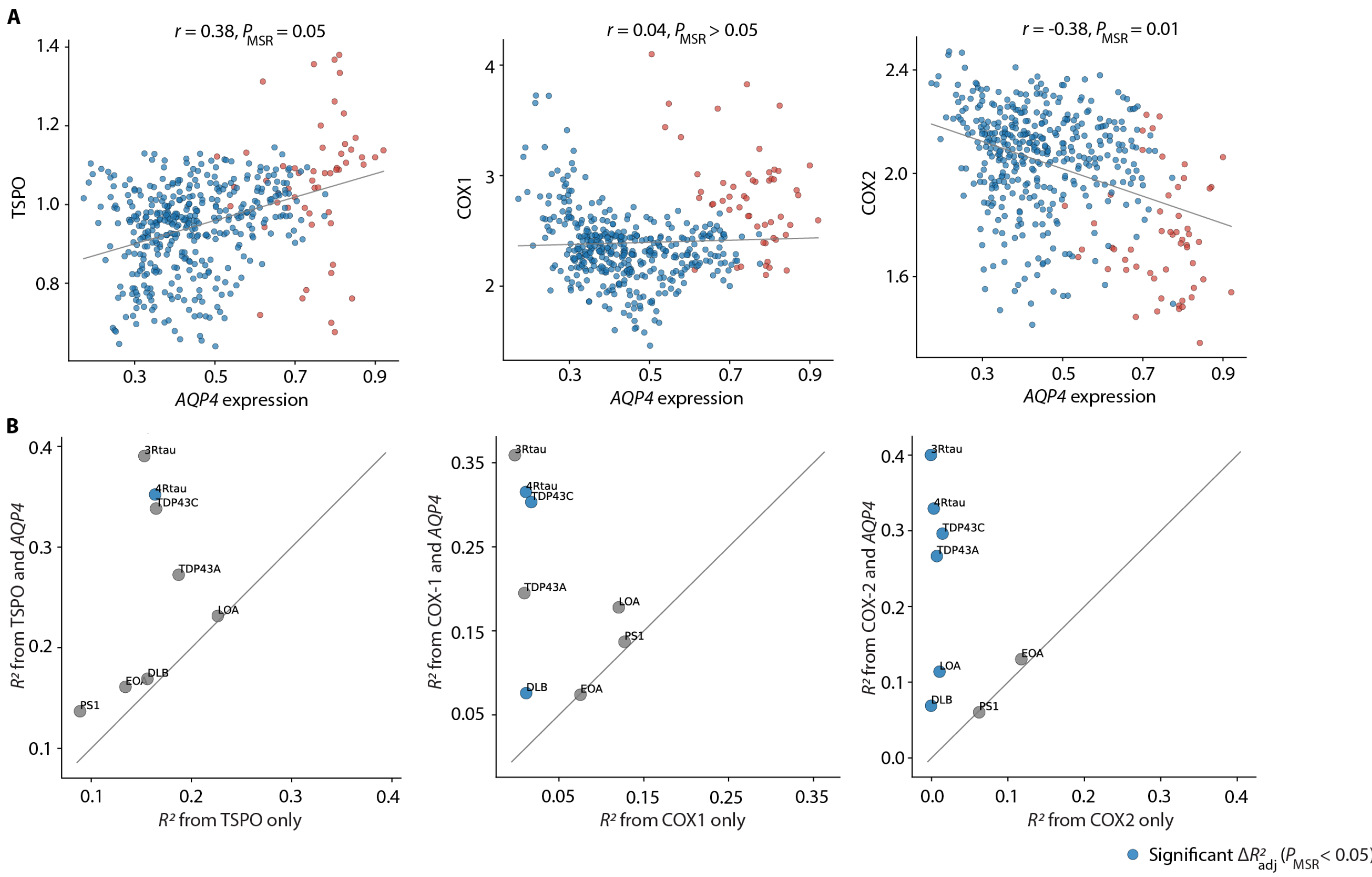

Figure S7. **Inflammatory markers and AQP4 expression predict neurodegenerative disease atrophy. |** (**A**) Spatial association between *AQP4* expression and inflammatory markers, including TSPO *(79)*, COX-1 *(80)*, and COX-2 *(81)* (from left to right). (**B**) For each disease, we quantify model fit for an inflammatory marker-only model (x-axis) and for a model that additionally includes AQP4 (y-axis), using adjusted R^2 (R^2_{adj}). Each point is a disease; the identity line is shown in grey. Points above the line indicate improved fit after adding AQP4. Orange points indicate significant improvements in fit relative to spatial autocorrelation–preserving null models generated with Moran spectral randomization ($p_{\mathrm{MSR}} < 0.05$) *(66)*. Disease abbreviations match Fig. 3

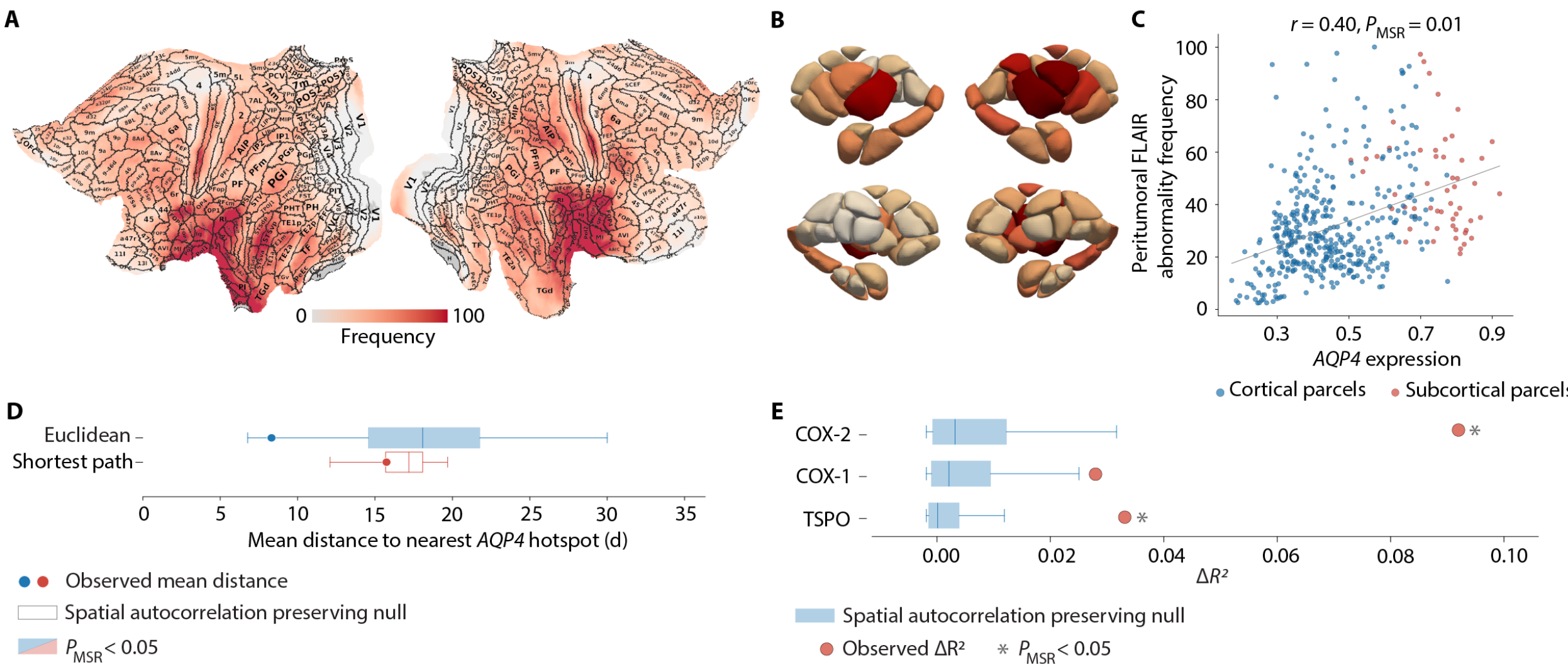

Figure S8. **Peritumoral Flair abnormality aligns with *AQP4* expression |** (**A,B**) Normative maps of peritumoral Flair abnormality frequency estimated from expert tumor delineations in two pre-operative glioma MRI cohorts ($N = 501$ *(88)* and $N = 630$ *(89)*; see *Methods*). Individual tumor masks are aggregated into a voxel-wise peritumoral Flair abnormality frequency map and parcellated to the Schaefer–Melbourne atlas *(25, 50)*. (**A**) Cortical peritumoral Flair abnormality frequency is shown on 2D flat fsLR surfaces with Glasser borders and areal abbreviations overlaid *(26)* (Table S2). (**B**) Subcortical peritumoral Flair abnormality frequency is shown in the Melbourne Subcortex S4 Atlas *(25)* (Table S1; Fig. S10). (**C**) Pearson's correlation between *AQP4* expression and peritumoral Flair abnormality frequency ($r = 0.40$, $p_{\mathrm{MSR}} = 0.01$). (**D**) Proximity of high-peritumoral Flair abnormality parcels (top decile of peritumoral Flair abnormality frequency) to *AQP4* hotspots (top decile of *AQP4* expression), quantified as mean Euclidean distance and mean shortest path distance along the SC. Shortest path distances are computed on a group-averaged connectome derived from the HCP S1200 release ($N = 1065$; see *Methods*) *(78)*. Observed mean distances are shown as points relative to spatial autocorrelation-preserving null distributions shown as boxplots. (**E**) Incremental variance explained ($\Delta R^2_{\mathrm{adj}}$) when adding TSPO *(79)*, COX-1 *(80)*, and COX-2 *(81)* to an *AQP4*-only model predicting peritumoral Flair abnormality frequency. Points show observed $\Delta R^2_{\mathrm{adj}}$ values, and boxplots show spatial autocorrelation–preserving null distributions; asterisks denote $p_{\mathrm{MSR}} < 0.05$.

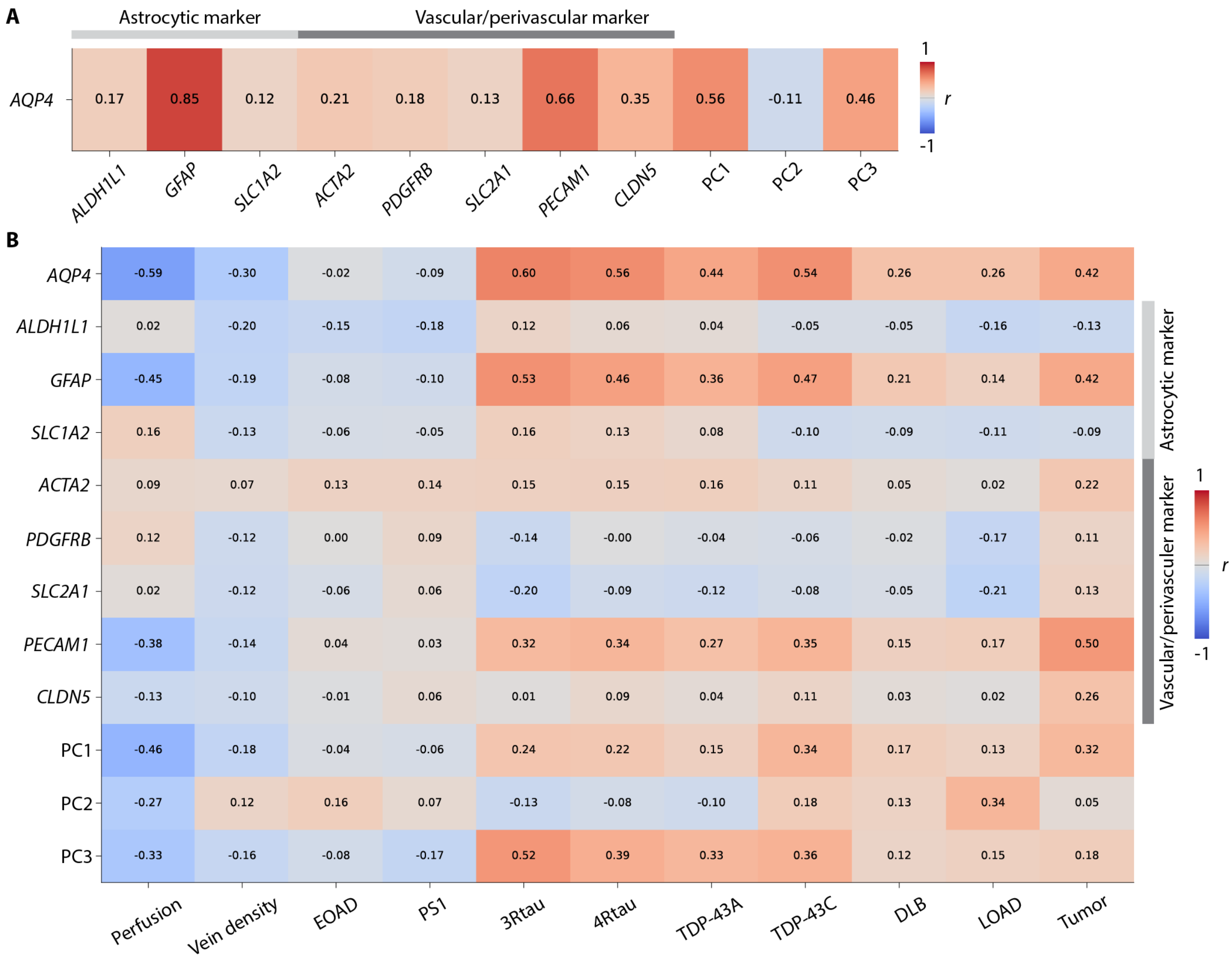

Figure S9. ***AQP4* associations are not fully reproduced by other marker maps** | (**A**) Pearson correlations between AHBA-derived *AQP4* expression and astrocyte-related markers, vascular/perivascular markers, and the first three principal components of gene expression (PC1, PC2, and PC3). (**B**) Pearson correlations between each marker map and the phenotypes examined in this study, including vascular measures, neurodegenerative atrophy patterns, and tumor frequency. Disease abbreviations match Fig. 3. Gene maps are processed with the `abagen` workflow *(24)* and parcellated to the Schaefer–Melbourne atlas (see *Methods*) *(25, 50)*.

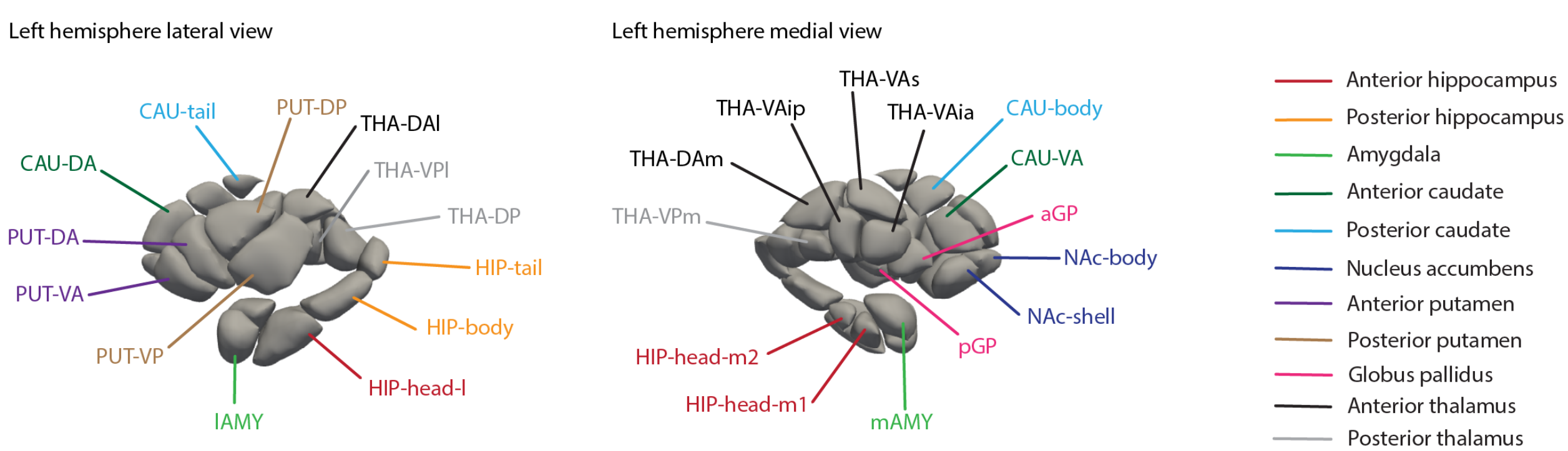

Figure S10. **Labels for Melbourne subcortical S4 parcels** | Left hemisphere lateral (left) and medial (right) views of the Melbourne subcortical S4 atlas *(25)* showing the parcel abbreviations. Abbreviations and full region names are listed in Table S1.

Abbreviation	Area's full name	Structure
HIP-head-m1	Hippocampus head, medial division - Subdivision 1	Anterior hippocampus
HIP-head-m2	Hippocampus head, medial division - Subdivision 2	Anterior hippocampus
HIP-head-l	Hippocampus head, lateral division	Anterior hippocampus
HIP-body	Hippocampus body	Posterior hippocampus
HIP-tail	Hippocampus tail	Posterior hippocampus
LAMY	Lateral amygdala	Amygdala
mAMY	Medial amygdala	Amygdala
CAU-DA	Dorsoanterior caudate	Anterior caudate
CAU-VA	Ventroanterior caudate	Anterior caudate
CAU-tail	Caudate tail	Posterior caudate
CAU-body	Caudate body	Posterior caudate
NAc-shell	Nucleus accumbens, shell	Nucleus accumbens
NAc-body	Nucleus accumbens, body	Nucleus accumbens
PUT-DA	Dorsoanterior putamen	Anterior putamen
PUT-VA	Ventroanterior putamen	Anterior putamen
PUT-DP	Dorsoposterior putamen	Posterior putamen
PUT-VP	Ventroposterior putamen	Posterior putamen
aGP	Anterior globus pallidus	Globus pallidus
pGP	Posterior globus pallidus	Globus pallidus
THA-DAl	Lateral dorsoanterior thalamus	Anterior thalamus
THA-DAm	Medial dorsoanterior thalamus	Anterior thalamus
THA-VAs	Superior ventroanterior thalamus	Anterior thalamus
THA-VAia	Inferior ventroanterior thalamus - Anterior division	Anterior thalamus
THA-VAip	Inferior ventroanterior thalamus - Posterior division	Anterior thalamus
THA-DP	Dorsoposterior thalamus	Posterior thalamus
THA-VPm	Medial ventroposterior thalamus	Posterior thalamus
THA-VPl	Lateral ventroposterior thalamus	Posterior thalamus

TABLE S1. **Nomenclature for the Melbourne subcortical S4 atlas *(25)* |** These labels are displayed in Fig. S10.

Abbreviation	Area's full name	Abbreviation	Area's full name
V1	Primary Visual Cortex	MST	Medial Superior Temporal Area
V6	Sixth Visual Area	V2	Second Visual Area
V3	Third Visual Area	V4	Fourth Visual Area
V8	Eighth Visual Area	4	Primary Motor Cortex
3b	Primary Sensory Cortex	FEF	Frontal Eye Fields
PEF	Premotor Eye Field	55b	Area 55b
V3A	Area V3A	RSC	RetroSplenial Complex
POS2	Parieto-Occipital Sulcus Area 2	V7	Seventh Visual Area
IPS1	IntraParietal Sulcus Area 1	FFC	Fusiform Face Complex
V3B	Area V3B	LO1	Area Lateral Occipital 1
LO2	Area Lateral Occipital 2	PIT	Posterior InferoTemporal complex
MT	Middle Temporal Area	A1	Primary Auditory Cortex
PSL	PeriSylvian Language Area	SFL	Superior Frontal Language Area
PCV	PreCuneus Visual Area	STV	Superior Temporal Visual Area
7Pm	Medial Area 7P	7m	Area 7m
POS1	Parieto-Occipital Sulcus Area 1	23d	Area 23d
v23ab	Area ventral 23 a+b	d23ab	Area dorsal 23 a+b
31pv	Area 31p ventral	5m	Area 5m
5mv	Area 5m ventral	23c	Area 23c
5L	Area 5L	24dd	Dorsal Area 24d
24dv	Ventral Area 24d	7AL	Lateral Area 7A
SCEF	Supplementary and Cingulate Eye Field	6ma	Area 6m anterior
7Am	Medial Area 7A	7Pl	Lateral Area 7P
7PC	Area 7PC	LIPv	Area Lateral IntraParietal ventral
VIP	Ventral IntraParietal Complex	MIP	Medial IntraParietal Area
1	Area 1	2	Area 2
3a	Area 3a	6d	Dorsal area 6
6mp	Area 6mp	6v	Ventral Area 6
p24pr	Area Posterior 24 prime	33pr	Area 33 prime
a24pr	Anterior 24 prime	p32pr	Area p32 prime
a24	Area a24	d32	Area dorsal 32
8BM	Area 8BM	p32	Area p32
10r	Area 10r	47m	Area 47m
8Av	Area 8Av	8Ad	Area 8Ad
9m	Area 9 Middle	8BL	Area 8B Lateral
9p	Area 9 Posterior	10d	Area 10d
8C	Area 8C	44	Area 44
45	Area 45	47l	Area 47l (47 lateral)
a47r	Area anterior 47r	6r	Rostral Area 6
IFJa	Area IFJa	IFJp	Area IFJp
IFSp	Area IFSp	IFSa	Area IFSa
p9-46v	Area posterior 9-46v	46	Area 46
a9-46v	Area anterior 9-46v	9-46d	Area 9-46d
9a	Area 9 anterior	10v	Area 10v
a10p	Area anterior 10p	10pp	Polar 10p
11l	Area 11l	13l	Area 13l
OFC	Orbital Frontal Complex	47s	Area 47s
LIPd	Area Lateral IntraParietal dorsal	6a	Area 6 anterior
i6-8	Inferior 6-8 Transitional Area	s6-8	Superior 6-8 Transitional Area
43	Area 43	OP4	Area OP4/PV
OP1	Area OP1/SII	OP2-3	Area OP2-3/VS
52	Area 52	RI	RetroInsular Cortex
PFcm	Area PFcm	PoI2	Posterior Insular Area 2
TA2	Area TA2	FOP4	Frontal OPercular Area 4

Abbreviation	Area's full name	Abbreviation	Area's full name
MI	Middle Insular Area	Pir	Piriform Cortex
AVI	Anterior Ventral Insular Area	AAIC	Anterior Agranular Insula Complex
FOP1	Frontal OPercular Area 1	FOP3	Frontal OPercular Area 3
FOP2	Frontal OPercular Area 2	PFt	Area PFt
AIP	Anterior IntraParietal Area	EC	Entorhinal Cortex
PreS	PreSubiculum	H	Hippocampus
ProS	ProStriate Area	PeEc	Perirhinal Ectorhinal Cortex
STGa	Area STGa	PBelt	ParaBelt Complex
A5	Auditory 5 Complex	PHA1	ParaHippocampal Area 1
PHA3	ParaHippocampal Area 3	STSda	Area STSd anterior
STSdp	Area STSd posterior	STSvp	Area STSv posterior
TGd	Area TG dorsal	TE1a	Area TE1 anterior
TE1p	Area TE1 posterior	TE2a	Area TE2 anterior
TF	Area TF	TE2p	Area TE2 posterior
PHT	Area PHT	PH	Area PH
TPOJ1	Area TemporoParietoOccipital Junction 1	TPOJ2	Area TemporoParietoOccipital Junction 2
TPOJ3	Area TemporoParietoOccipital Junction 3	DVT	Dorsal Transitional Visual Area
PGp	Area PGp	IP2	Area IntraParietal 2
IP1	Area IntraParietal 1	IP0	Area IntraParietal 0
PFop	Area PF opercular	PF	Area PF Complex
PFm	Area PFm Complex	PGi	Area PGi
PGs	Area PGs	V6A	Area V6A
VMV1	VentroMedial Visual Area 1	VMV3	VentroMedial Visual Area 3
PHA2	ParaHippocampal Area 2	V4t	Area V4t
FST	Area FST	V3CD	Area V3CD
LO3	Area Lateral Occipital 3	VMV2	VentroMedial Visual Area 2
31pd	Area 31pd	31a	Area 31a
VVC	Ventral Visual Complex	25	Area 25
s32	Area s32	pOFC	Posterior OFC Complex
PoI1	Area Posterior Insular 1	Ig	Insular Granular Complex
FOP5	Area Frontal Opercular 5	p10p	Area posterior 10p
p47r	Area posterior 47r	TGv	Area TG Ventral
MBelt	Medial Belt Complex	LBelt	Lateral Belt Complex
A4	Auditory 4 Complex	STSva	Area STSv anterior
TE1m	Area TE1 Middle	PI	Para-Insular Area
a32pr	Area anterior 32 prime	p24	Area posterior 24

TABLE S2. **Nomenclature for the multi-modal Glasser parcellation *(26)*.**
